# Core Fucosylation Represses SMURF1‐Dependent Degradation of CD47 to Promote Tumor Immune Evasion

**DOI:** 10.1002/advs.202516863

**Published:** 2025-12-07

**Authors:** Yuting Cao, Siyuan Chai, Mingyang Li, Xiaoming Chen, Jiating Hu, Bingyi Lin, Liming Wu, Wen Yi, Qiang Zhu

**Affiliations:** ^1^ Department of Biochemistry College of Life Sciences Zhejiang University Hangzhou 310058 China; ^2^ Department of Hepatobiliary and Pancreatic Surgery The First Affiliated Hospital Zhejiang Provincial Key Laboratory of Pancreatic Disease School of Medicine Zhejiang University Hangzhou 310003 China; ^3^ School of Laboratory Medicine and Life Sciences Wenzhou Medical University Wenzhou 325035 China

**Keywords:** CD47, core fucosylation, liver cancer, tumor immunity

## Abstract

Glycosylation, the covalent attachment of glycans to proteins, lipids, and RNAs, is fundamental in regulating diverse biological processes. Glycosylation patterns are aberrantly altered in the tumor microenvironment and closely associated with tumor immune escape. However, the molecular mechanisms by which glycosylation regulates tumor immune escape are poorly understood. We show that Cluster of Differentiation 47 (CD47), an innate immune checkpoint protein, is highly modified with core fucosylated N‐linked glycans. Core fucosylation of CD47 mediated by fucosyltransferase 8 (FUT8) at asparagine 111 (N111) reduces CD47 ubiquitination and degradation. Blockade of N111 glycosylation represses CD47 expression and promotes macrophage phagocytosis of tumor cells. Furthermore, elimination of N111 glycosylation promotes the infiltration of CD103^+^ dendritic cells (DCs), leading to the increased recruitment of natural killer (NK) cells and inhibition of tumor growth in a murine hepatocellular carcinoma (HCC) model. Combined treatment with core fucosylation inhibitors and an anti‐CD47 antibody synergistically promotes therapeutic efficacy in the HCC model. Finally, FUT8 levels in human HCC specimens are positively correlated with CD47 expressions and negatively correlated with the infiltration of CD103^+^ DC and NK cells. Collectively, this study reveals a mechanism underlying CD47 upregulation in tumor cells and highlights the potential of targeting the FUT8‐SMURF1‐CD47 axis as a therapeutic strategy to improve anti‐tumor immune responses.

## Graphical abstract

FUT8‐mediated core fucosylation of CD47 at N111 blocks SMURF1 binding and reduces CD47 ubiquitination and degradation. Blocking N111 glycosylation reduces CD47 expression and promotes macrophage phagocytosis of tumor cells. Furthermore, ablating CD47 core fucosylation boosts CD103+ dendritic cells (DCs) infiltration, increases natural killer (NK) cell recruitment, and suppresses tumor growth.

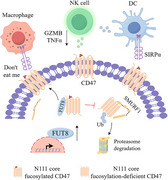

## 1 Introduction

Glycosylation is the most prevalent and complex form of post‐translational modification (PTM), which plays a critical role in regulating diverse physiological processes ranging from cell‐cell communication, cellular signaling, to cell adhesion and migration.^[^ ^1^ ^]^ During tumor development and progression, glycosylation patterns (including glyco‐forms and expression levels) on tumor cells are aberrantly altered.^[^ ^2^ ^]^ Studies have demonstrated that altered glycosylation facilitates uncontrolled cell growth, tumor invasion and metastasis, and angiogenesis.^[^ ^3^, ^4^, ^5^, ^6^ ^]^ Recent research has shown that altered glycosylation also critically influences the interactions between tumor and immune cells in the tumor microenvironment (TME) to modulate immune cell activities.^[^ ^7^ ^]^ Altered glycosylation is an integral part of the TME that contributes to tumor growth by endowing tumor cells with the ability to escape immune cell surveillance and destruction.^[^ ^8^ ^]^ The altered glycosylation serves as a tumor‐specific “glyco‐code” that suppresses the immune responses and impairs the efficacy of immunotherapy.^[^ ^9^, ^10^ ^]^ Thus, understanding the function of specific glycosylation in regulating tumor immunity is critical to designing more effective immunotherapies.

The innate immune system serves as the primary defense mechanism against infections and tumor development.^[^ ^11^ ^]^ Antigen‐presenting cells (APCs), such as monocytes, dendritic cells (DCs), and macrophages, play a crucial role in establishing phagocytosis during the innate immune response that captures and eliminates malignant cells.^[^ ^12^ ^]^ However, tumors often develop immune resistance by upregulating immune checkpoint proteins to dampen the immune response.^[^ ^13^ ^]^ Cluster of differentiation 47 (CD47), a glycoprotein frequently upregulated in tumor cells, has been shown to play an important role in mediating innate immune evasion.^[^ ^14^, ^15^, ^16^ ^]^ Mechanistically, CD47 interacts with the Signal‐Regulatory Protein alpha (SIRPα) receptor on myeloid cells to repress phagocytosis.^[^ ^17^, ^18^ ^]^ Based on this mechanism, several clinical studies are undergoing that employ anti‐CD47 antibodies to block SIRPα‐CD47 interaction and augment the efficacy of immunotherapy in patients with hematologic,^[^ ^19^ ^]^ head and neck,^[^ ^20^ ^]^ colorectal,^[^ ^21^ ^]^ endometrial,^[^ ^22^ ^]^ ovarian,^[^ ^23^ ^]^ hepatocellular,^[^ ^24^ ^]^ non‐small cell lung,^[^ ^25^ ^]^ and HER2^+^gastroesophageal cancers.^[^ ^26^, ^27^ ^]^ However, CD47 is also expressed on healthy cells such as red blood cells (RBCs) and thrombocytes.^[^ ^23^, ^26^ ^]^ Protein‐centric targeting strategies consequently lead to “off‐target effect” and limit the impact on tumor growth. Therefore, it is imperative to further investigate the regulatory mechanisms of CD47 and identify new targets in the CD47‐SIRPα signaling axis.

CD47 expression can be modulated at both transcriptional and translational levels.^[^ ^28^ ^]^ A number of transcription factors are shown to regulate CD47 transcription activation, including Signal Transducer and Activator of Transcription 3 (STAT3),^[^ ^29^ ^]^ β‐catenin‐transcription Factor 4 (TCF4),^[^ ^30^ ^]^ Hypoxia‐Inducible Factor 1 (HIF‐1),^[^ ^31^ ^]^ c‐Myc,^[^ ^32^ ^]^ and SREBP1.^[^ ^15^ ^]^ PTMs are also involved in the regulation of CD47 protein expression by modulating its cellular degradation. For example, c‐Src‐induced CD47 Y288 phosphorylation repressed the binding of the ubiquitin E3 ligase TRIM21 with CD47, leading to reduced degradation and increased expression of CD47.^[^ ^33^ ^]^ TRAF2 induced the ubiquitination of the C‐terminal of CD47, and repressed CD47 autophagic degradation by inhibiting its binding to LC3.^[^ ^14^ ^]^ CD47 has been identified as a glycoprotein, but the role of CD47 glycosylation in regulating its interaction with SIRPα remains controversial. While earlier investigations showed that CD47 glycosylation did not influence its binding to SIRPα,^[^ ^34^ ^]^ a more recent finding demonstrated that glycosylation enhanced the CD47‐SIRPα interaction.^[^ ^35^ ^]^ Despite these studies, whether glycosylation regulates CD47 expression remains to be elucidated.

Core fucosylation, a prevalent type of N‐linked glycosylation catalyzed by fucosyltransferase 8 (FUT8), is defined as the attachment of L‐fucose to the innermost N‐acetyl‐D‐glucosamine (GlcNAc) residue of N‐linked glycans via an α‐1,6 linkage.^[^ ^36^ ^]^ Core fucosylation is upregulated in various types of cancer and plays a pivotal role in tumor malignancy, including immune evasion.^[^ ^37^, ^38^, ^39^ ^]^ Recent studies have demonstrated that core fucosylation can modulate adaptive immunity by modifying immune checkpoint proteins.^[^ ^37^, ^40^ ^]^ For instance, Huang et al. showed that core fucosylation of B7H3 hinders its interaction with ubiquitin ligases, increasing the stability of B7H3 and contributing to immune evasion in triple‐negative breast cancers (TNBC).^[^ ^37^ ^]^ Core fucosylation also occurs on the PD‐1 protein expressed in T cells.^[^ ^40^ ^]^ Blockade of core fucosylation reduces PD‐1 expression in T cells, disrupts the interaction between PD‐1 and PD‐L1, leading to T cell activation and enhancement of the tumor killing efficiency.^[^ ^40^ ^]^ Similar to PD‐1, both PD‐L1 and PD‐L2 possess core fucosylation. Core fucosylation stabilizes PD‐L2 expression by inhibiting its lysosomal degradation pathway, enhances its binding to PD‐1, and facilitates tumor immune escape.^[^ ^41^, ^42^ ^]^ Despite these advances in adaptive immunity, the role of core fucosylation in innate immunity remains elusive.

In this study, we demonstrate that FUT8‐mediated core fucosylation of CD47 at asparagine 111 (N111) impaired its association with the ubiquitin E3 ligase SMURF1, resulting in abrogating SMURF1‐mediated CD47 K85/99/102 polyubiquitylation and CD47 degradation. Reconstituted expression of the N111Q CD47 mutant increased the infiltration and activity of CD103^+^ DCs to enhance the macrophage phagocytosis of tumor cells. This, in turn, increased the recruitment of NK cells and NK‐mediated tumor cell killing, leading to the suppression of liver cancer growth in mice. Notably, the core fucosylation inhibitor 2F‐Fuc synergized with the anti‐CD47 antibody to enhance the therapeutic efficacy of liver cancer in mouse models. Collectively, our study identifies a previously unknown mechanism regulating CD47 protein stability and further suggests a new strategy to enhance liver cancer immunotherapy by targeting CD47 core fucosylation.

## 2 Results

### 2.1 CD47 Contains Core Fucosylated N‐Glycans

**Figure 1:**
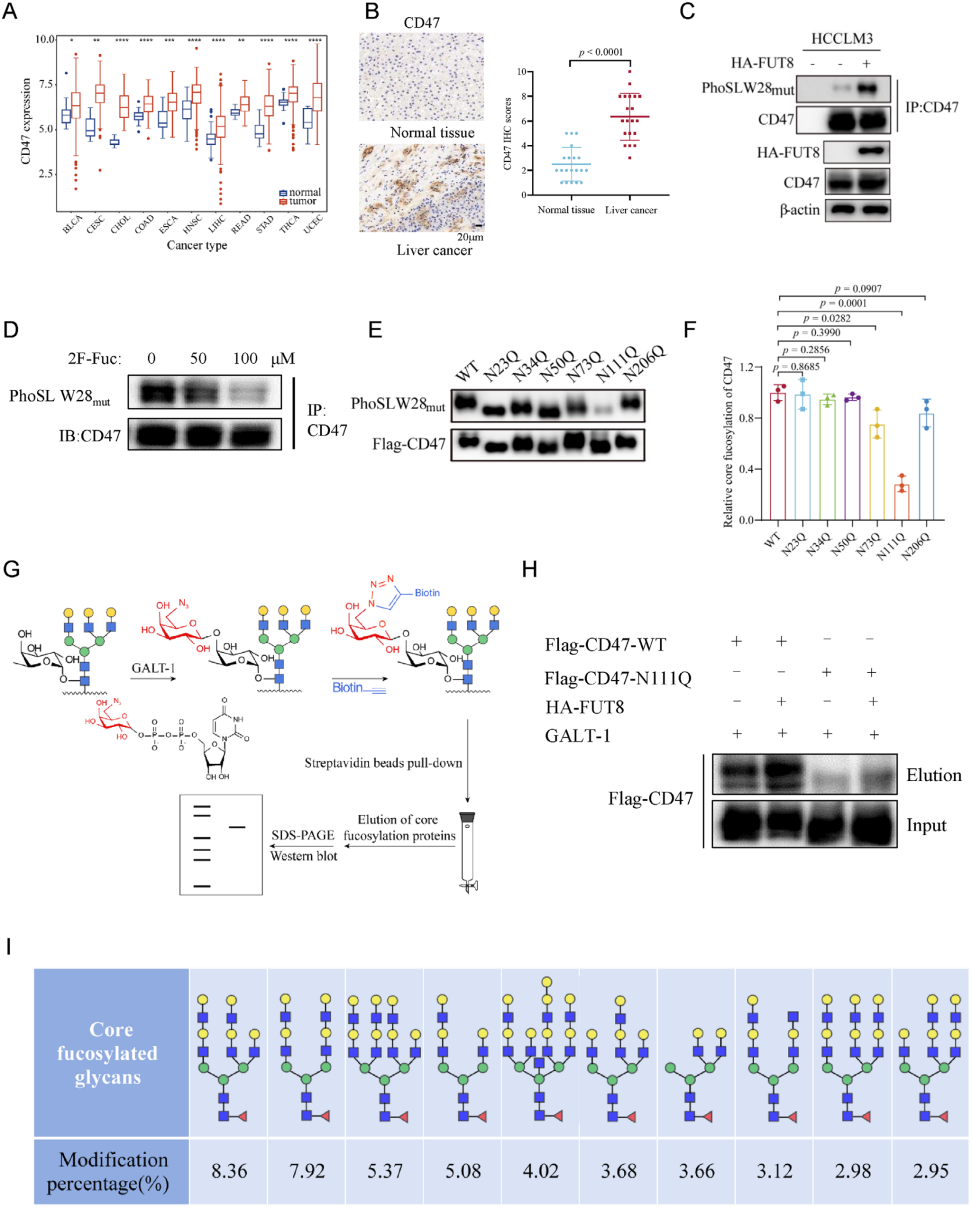
CD47 contains core fucosylated N‐glycans. A) The mRNA expression of CD47 in various cancers and corresponding normal tissues from the TCGA and GTEx databases. B) Immunohistochemistry analysis of CD47 expression in normal tissue and liver cancer. (Scale bar, 20 µm) C) Lectin blotting of CD47 with PhoSLW28_mut_ in HCCLM3 cells expressing control vector or HA‐FUT8. D) Lectin blotting of CD47 with PhoSLW28_mut_ in HCCLM3 cells upon different concentrations of 2F‐Fuc treatment (0, 50, 100 µm). E) Lectin blotting of Flag‐CD47 WT and corresponding glycosylation mutants with PhoSLW28_mut_ in 293T cells. F) Quantification of core fucosylation levels of CD47 WT and mutants. n = 3; Data are depicted as means ± SD. P‐values were calculated by unpaired two‐tailed Student's t‐tests. G) Scheme of detecting protein core fucosylation level by chemoenzymatic labeling method. H) Immunoblotting of core fucosylation levels of immunoprecipitated exogenous Flag‐CD47 WT and N111Q in 293T cells expressing control vector or HA‐FUT8 by chemoenzymatic labeling approach. I) RPLC‐MS/MS of the top ten N‐glycans on the N111 site of purified human CD47 protein.

CD47 expression has been shown to be upregulated in several types of cancers, including colorectal,^[^ ^43^ ^]^ gastric,^[^ ^44^ ^]^ liver,^[^ ^45^ ^]^ lung,^[^ ^46^ ^]^ and acute myeloid leukemia.^[^ ^47^ ^]^ To corroborate these findings, we analyzed CD47 mRNA expressions in tumor tissues and normal tissues based on The Cancer Genome Atlas (TCGA) gene expression datasets. The results showed that CD47 mRNA expressions were markedly upregulated in multiple cancers (**Figure** **1**A). Consistently, further analyses of human hepatocellular carcinoma (HCC) tissues and adjacent matched normal tissues with Western blotting and immunohistochemical (IHC) revealed that CD47 protein expression levels were significantly higher in cancer tissues (Figures 1B; S1A, Supporting Information). The Kaplan‐Meier analysis also demonstrated that high levels of CD47 mRNA expression were correlated with poor prognosis of patients with PAAD, ESCA, LIHC, and Lung Squamous Cell Carcinoma (LUSC) (Figure S1B, Supporting Information). These data indicate that CD47 expression is closely associated with tumor development.

CD47 is a glycoprotein residing on the cell surface. To determine the glycosylation pattern of CD47, we treated human liver cancer cell lines MHCC97‐H and HCCLM3 cell lysates with the recombinant peptide‐N‐glycosidase F (PNGase F), which can cleave N‐glycans, and O‐glycosidase that can remove O‐glycans. The results showed that a notable band shift of CD47 from ≈55 to ≈37 kDa occurred upon PNGase F treatment, whereas O‐glycosidase treatment had no effect (Figure S1C,D, Supporting Information). Furthermore, CD47 glycosylation was dramatically inhibited in cells upon treatment with N‐linked glycosylation inhibitor tunicamycin (TM), but not O‐glycosidase inhibitors Thiamet G (TMG) or PUGNAc, suggesting that CD47 mainly possesses N‐glycans (Figure S1E,F, Supporting Information). To further determine the specific N‐glycosylation sites of CD47, we mutated the asparagine (N) residues in all of the N‐X‐T motifs (the consensus sequence of N‐glycosylation) to glutamine (Q) individually to generate potential N‐glycan‐deficient mutants. We found that, except for N206Q, all other singlet mutants (N23Q, N34Q, N50Q, N73Q, and N111Q) displayed a noticeable decrease in molecular weight as compared to the WT (Figure S1G, Supporting Information). In addition, the quintuple mutant in which N23, N34, N50, N73, and N111 were simultaneously mutated to Q (referred to as 5NQ), completely ablated CD47 glycosylation similar to that after treatment with PNGase F, suggesting that N‐glycans are present at these five sites (Figure S1H, Supporting Information).

Core fucosylation is commonly found in N‐glycans and is closely associated with tumor development and progression.^[^ ^48^ ^]^ To investigate whether CD47 N‐glycosylation possesses core fucosylation, we employed two lectins, Lens culinaris agglutinin (LCA) and engineered Pholiota squarrosa lectin (PhoSL W28_mut_),^[^ ^49^ ^]^ which specifically recognize core fucosylation. CRISPR‐Cas9‐mediated knockout of FUT8 in 293T cells almost abolishes CD47 core fucosylation signals detected by PhoSL W28_mut_, confirming the high specificity of this lectin blotting (Figure S1I, Supporting Information). Ectopic expression of FUT8 significantly elevated the blotting signal of CD47 core fucosylation (Figure 1C). Consistently, treatment with a fucosylation inhibitor 2‐fluoro‐L‐fucose (2F‐Fuc) significantly reduced the blotting signals (Figure 1D). We also observed that the core fucosylation signal showed a drastic reduction in N111Q mutant, but not other mutants, compared to the WT (Figure 1E,F). As another verification, we employed a highly selective chemoenzymatic labeling approach to analyze CD47 core fucosylation.^[^ ^50^ ^]^ Cell lysates from MHCC97‐H cells were enzymatically labeled with an azido‐appended galactose residue with a specific galactosyltransferase (GalT1) derived from *Caenorhabditis elegans*. The resulting azido‐labeled proteins were then coupled with an alkyne‐containing biotin reporter via copper(I)‐mediated azide–alkyne cycloaddition (CuAAC) chemistry. The biotinylated proteins were subsequently captured with streptavidin beads, eluted, and analyzed by immunoblotting using the CD47 antibody (Figure 1G). In line with results by lectin blotting, WT CD47 displayed a higher immunoblotting signal compared to the N111Q mutant (Figures 1H; S1J, Supporting Information). FUT8 overexpression increased the core fucosylation signal of WT CD47, but not the N111Q mutant (Figures 1H; S1J, Supporting Information). To further profile the detailed glycan structures at N111, we analyzed the digested peptides of purified CD47 expressed in 293T cells by Reversed‐Phase Liquid Chromatography‐Mass Spectrometry (RPLC‐MS/MS). We identified a total of 271 intact N‐glycopeptides at N111, among which 88 contained core fucose. The majority of glycans containing core fucose are of the complex and hybrid types (Figures 1I; S2, Supporting Information). Together, these results demonstrate that N111 of CD47 possesses core fucosylated glycans.

### 2.2 Core Fucosylation on N111 Represses Proteasomal Degradation of CD47

**Figure 2:**
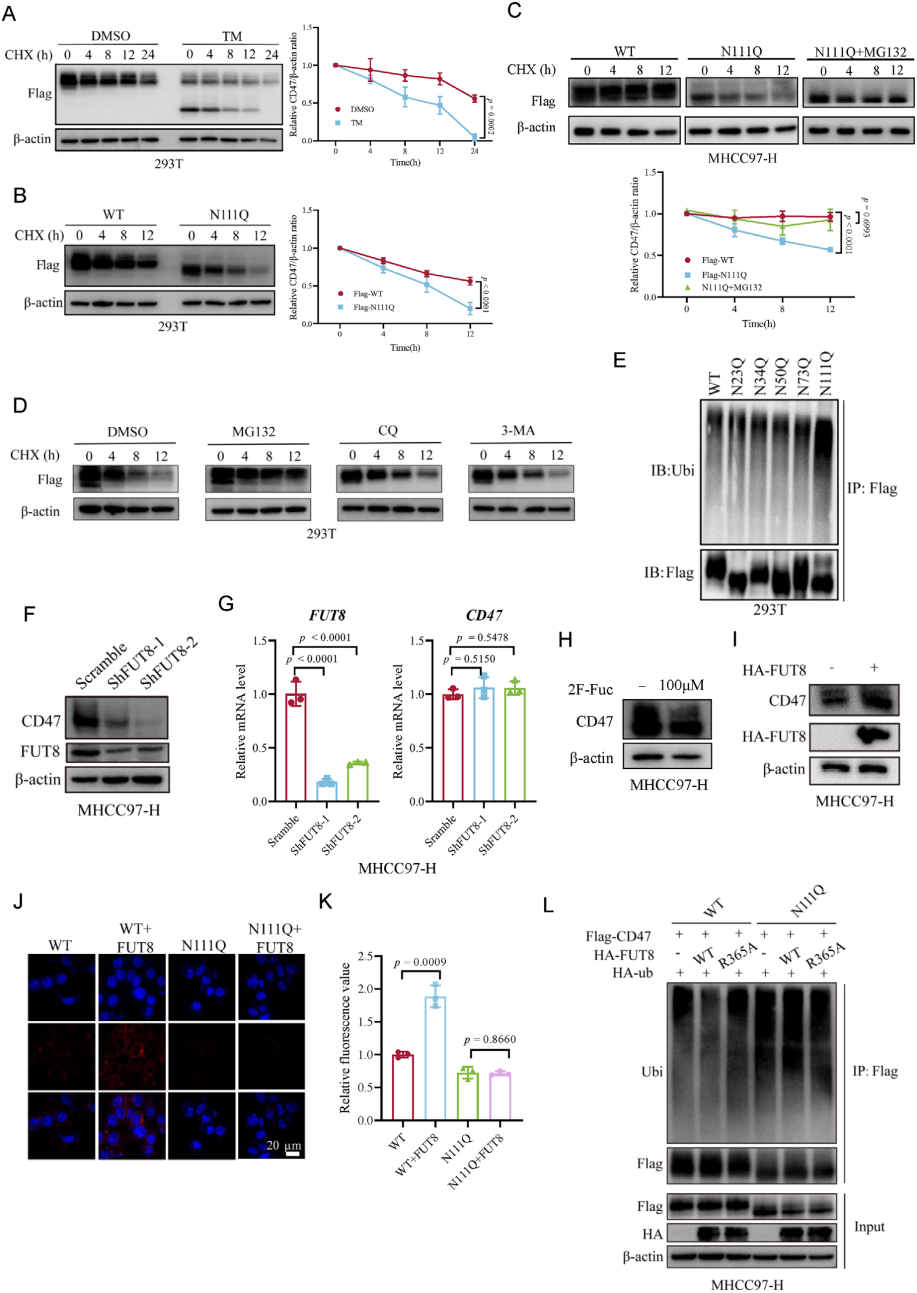
Core fucosylation on N111 represses proteasomal degradation of CD47. A) Immunoblotting of CD47 levels in 293T cells by CHX treatment in the presence of DMSO or TM. Quantification was shown. n = 3; Data are presented as means ± SD. P values were determined by unpaired two‐tailed Student's t tests. B) Immunoblotting of CD47 levels in 293T cells overexpressing Flag‐tagged WT and N111Q mutant by CHX treatment. Quantification was shown. n = 3; Data are presented as means ± SD. P values were determined by unpaired two‐tailed Student's t tests. C) Immunoblotting of CD47 levels in WT or N111Q reconstituted MHCC97‐H cells by CHX treatment in the presence of DMSO or MG132. Quantification was shown. n = 3; Data are presented as means ± SD. P values were determined by unpaired two‐tailed Student's t tests. D) Immunoblotting of CD47 levels in 293T cells overexpressing the N111Q mutant by CHX treatment in the presence of DMSO, MG132, CQ, and 3‐MA. E) Immunoblotting of ubiquitination levels of CD47 WT and mutants. F) Immunoblotting of CD47 protein in MHCC97‐H cells expressing scramble or shFUT8. G) Quantitative PCR analysis of FUT8 and CD47 mRNA levels in MHCC97‐H cells expressing scramble or shFUT8. n = 3; P values were determined by unpaired two‐tailed Student's t tests. H) Immunoblotting of CD47 protein in MHCC97‐H cells treated with DMSO or 100 µm 2F‐Fuc. I) Immunoblotting of CD47 protein in MHCC97‐H cells expressing a control vector or HA‐FUT8. J, K) Immunofluorescence analysis of CD47 protein expression on the cell membrane in CD47 WT or N111Q reconstituted MHCC97‐H cells expressing control vector or HA‐FUT8 (J). Relative fluorescence value was measured (K). n = 3; (Scale bar, 20 µm). P values were determined by unpaired two‐tailed Student's t tests. L) Immunoblotting of ubiquitination levels of CD47 in CD47 WT or N111Q reconstituted MHCC97‐H cells expressing vector, HA‐FUT8 WT, and HA‐FUT8 R365A.

Next, to probe the function of N‐glycosylation on CD47, we analyzed the interaction between CD47 and SIRPα. Consistent with the previous study,^[^ ^34^ ^]^ blockade of N‐glycosylation on CD47 via site mutations or TM treatment showed no apparent impact on the binding of CD47 and SIRPα (Figure S3A,B, Supporting Information). TM treatment reduced the protein expression but not the mRNA expression of CD47 in MHCC97‐H and HCCLM3 cells, suggesting that N‐glycosylation is important for CD47 stability (Figure S3C, Supporting Information). We then analyzed the half‐life of CD47 using protein synthesis inhibitor cycloheximide (CHX) and found that TM‐treated CD47 exhibited a faster turnover rate than DMSO‐treated CD47 (**Figure** **2**A). Notably, N111Q, but not other mutants, displayed a faster turnover rate than WT CD47 (Figures 2B; S3D,E, Supporting Information). We then generated stably transfected MHCC97‐H cells with depletion of the endogenous CD47, and reconstituted with shRNA‐resistant WT or N111Q CD47 (Figure S3F, Supporting Information). Consistently, the N111Q mutant attenuated CD47 protein expression and promoted the degradation of CD47 as compared to the WT CD47 (Figure 2C). These data indicated that N‐glycosylation of CD47 on N111 is responsible for CD47 stability.

To further understand which degradation pathway of CD47 is regulated by N111 glycosylation, we treated N111Q CD47 reconstituted MHCC97‐H cells with lysosomal inhibitor chloroquine (CQ), the proteasomal inhibitor MG132, or the autophagy inhibitor 3‐Methyladenine (3‐MA), and examined CD47 expression. The result showed that only MG132 could rescue the expression of CD47 (Figure 2D). Moreover, treatment with MG132 fully rescued the turn‐over rate of CD47 in N111Q CD47 reconstituted MHCC97‐H cells, manifesting that N111 glycosylation regulates CD47 degradation via the proteasomal pathway (Figure 2C). In line with this result, TM treatment elevated the ubiquitination level of CD47 (Figure S3G, Supporting Information). The N111Q mutant, but not other mutants, showed increased ubiquitination levels compared to the WT CD47 (Figure 2E). Similar results were obtained in Hepa1‐6 murine liver cancer cells in which endogenous CD47 was depleted and reconstituted with shRNA‐resistant WT or N109Q CD47 (the corresponding glycosylation site in mice) (Figure S3E,H, Supporting Information). To specifically verify the glycosylation site of CD47, we generated MHCC97‐H cells with reconstituted expression of the other two N111 mutants: N111A (deglycosylated but retaining the asparagine skeleton) and N111T (introducing potential O‐glycosylation competition). The CD47 expression levels and protein half‐lives of these mutants were comparable to those observed for the canonical N111Q mutant (Figure S3I,J, Supporting Information). Taken together, these results demonstrate that N111 glycosylation upregulates CD47 levels via repressing the proteasomal degradation of CD47.

As N111 possesses core fucosylation, we next probed the impact of core fucosylation on CD47 expression. We constructed *FUT8* knockdown HCC stable cell lines by short‐hairpin RNAs. The depletion of *FUT8* or treatment with 2F‐Fuc reduced, whereas overexpression of FUT8 increased, CD47 protein levels but not mRNA levels in MHCC97‐H and HCCLM3 cells (Figures 2F–I; S4A–D, Supporting Information). Given that CD47 is a transmembrane protein, we then analyzed its cell‐surface expression levels. Flow cytometry and immunofluorescence assays showed CD47 expression levels on the cell membrane were significantly reduced upon *FUT8* ablation (Figure S4E–H, Supporting Information). Consistently, overexpression of FUT8 elevated the cell‐surface expression of CD47 in WT, but not N111Q CD47, rescued MHCC97‐H cells (Figures 2J,K; S4I,J, Supporting Information). We next explored the effect of core fucosylation on the degradation of CD47. The depletion of *FUT8* enhanced, whereas overexpression of FUT8 reduced, the turnover rate of CD47 in WT, but not N111Q CD47, rescued MHCC97‐H cells (Figure S5A,B, Supporting Information). Treatment with MG132, but not CQ or 3‐MA, restored the reduced CD47 expression mediated by *FUT8* knockdown (Figure S5C,D, Supporting Information). Besides, overexpression of WT FUT8, but not the catalytically dead mutant (R365A), significantly repressed the ubiquitination level of WT CD47, whereas no apparent effect was observed on N111Q CD47 (Figures 2L; S5E, Supporting Information). Collectively, these data demonstrated that core fucosylation of CD47 at N111 inhibits ubiquitination‐mediated proteasomal degradation of CD47.

### 2.3 N111 Core Fucosylation Promotes CD47 Stability by Inhibiting the Binding of CD47 with SMURF1

**Figure 3:**
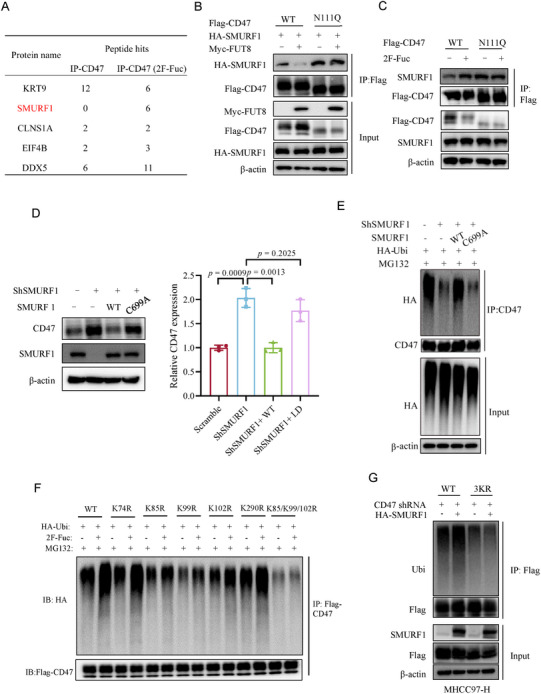
N111 core fucosylation promotes CD47 stability by inhibiting the binding of CD47 with SMURF1. A) Co‐IP‐MS analysis of endogenous CD47 in MHCC97‐H cells in the presence or absence of 2F‐Fuc treatment. B) Immunoblotting of CD47 and SMURF1 interaction in CD47 WT or N111Q reconstituted MHCC97‐H cells expressing vector and HA‐FUT8. C) Immunoblotting of CD47 and SMURF1 interaction in CD47 WT or N111Q reconstituted MHCC97‐H cells in the presence of DMSO and 2F‐Fuc. D) Immunoblotting of CD47 protein in MHCC97‐H cells expressing scramble, shSMURF1, SMURF1 WT, and C699A reconstituted MHCC97‐H cells. n = 3; Data are depicted as means ± SD. P‐values were calculated by unpaired two‐tailed Student's t‐tests. E) Immunoblotting of ubiquitination levels of CD47 in MHCC97‐H cells expressing shSMURF1, SMURF1 WT, and C699A reconstituted MHCC97‐H cells. F) Immunoblotting of ubiquitination levels of CD47 WT, K74R, K85R, K99R, K102R, and K290R in CD47‐depleted MHCC97‐H cells in the presence or absence of 2F‐Fuc treatment. G) Immunoblotting of ubiquitination levels of CD47 in CD47 WT and 3KR reconstituted MHCC97‐H cells expressing vector or HA‐SMURF1.

To further investigate the mechanism by which core fucosylation stabilized CD47 protein expression, we performed Co‐IP‐MS of endogenous CD47 in MHCC97‐H cells in the presence or absence of 2F‐Fuc treatment. The result showed that SMURF1, an E3 ubiquitin ligase, bound CD47 to a much greater extent upon 2F‐Fuc treatment, which was further validated with Co‐IP and immunofluorescence assays in MHCC97‐H cells (**Figures** **3**A; S6A,B, Supporting Information). Notably, FUT8 overexpression increased CD47 expression and reduced the binding of CD47 with SMURF1 (Figure 3B). Compared to the WT, the N111Q mutant exhibited consistent binding to SMURF1, which was resistant to 2F‐Fuc treatment or FUT8 expression‐mediated disruption of the SMURF1‐CD47 complex (Figure 3B,C). To clarify how N111 core fucosylation interferes with SMURF1‐CD47 interaction, we employed AlphaFold2 to predict the structure of the SMURF1‐CD47 complex, which revealed that the extracellular domain (ECD) of CD47 interacts with the N‐terminal C2 domain of SMURF1(Figure S6C, Supporting Information). This interaction was validated via co‐IP assays (Figure S6D, Supporting Information). We further performed all‐atom molecular dynamics simulations of this interaction with N111 in both nonglycosylated and glycosylated forms. The results revealed that N111 core fucosylation of CD47 inhibits the formation of hydrogen bonds at the SMURF1‐CD47 interface, thereby inhibiting their interaction (Figure S6E, Supporting Information).

To profile the regulation of potential immune‐related substrates by SMURF1, we performed quantitative proteomic analysis comparing global protein expression between control and SMURF1 knockdown MHCC97‐H cells. Across three biological replicates, we identified 7352 proteins with at least two unique peptides. Among these, the expression levels of 772 proteins exhibited significant up‐regulation, and 1196 proteins down‐regulation upon SMURF1 knockdown (fold change >2, *p* < 0.05) (Figure S6F, Supporting Information). Notably, the expression of CD47 was greatly up‐regulated in SMURF1‐depleted MHCC97‐H cells. We also analyzed other immune‐related substrates such as immune checkpoint molecules, interleukin receptors, and other TME‐associated modulators, as shown in Figure S6G (Supporting Information). The results revealed that the expression levels of these proteins did not exhibit significant elevation upon SMURF1 knockdown. Thus, we considered CD47 as the primary immune‐related substrate of SMURF1 in HCC cells. Consistently, depletion of SMURF1 increased CD47 expression and concomitantly decreased the ubiquitination of CD47 in MHCC97‐H cells (Figures 3D,E; S7A,B, Supporting Information). On the contrary, overexpression of SMURF1 reduced the expression of CD47 and enhanced the ubiquitination of CD47 (Figures 3D,E; S7A,B, Supporting Information). Reconstituted expression of WT, but not E3 ligase‐dead mutant (C699A) SMURF1, which was also verified by an in vitro ubiquitination assay, in SMURF1‐depleted MHCC97‐H cells enhanced CD47 ubiquitination and decreased CD47 levels (Figures 3D,E; S7C, Supporting Information). To explore the ubiquitin chain types involved in CD47 ubiquitination mediated by SMURF1, we co‐transfected HEK293T cells with Flag‐CD47 and cMyc‐SMURF1 alongside either wild‐type ubiquitin (Ub WT), lysine 48‐only ubiquitin (Ub K48O), or lysine 63‐only ubiquitin (Ub K63O) (Figure S7D, Supporting Information). Notably, robust CD47 ubiquitination was observed upon co‐transfection with Ub WT or Ub K48O, whereas Ub K63O co‐transfection failed to enhance ubiquitination. Moreover, the SMURF1‐induced polyubiquitination of CD47 was abolished when using the Ub K48R mutant (Figure S7D, Supporting Information). Collectively, these results suggest that FUT8‐mediated core fucosylation of CD47 at N111 represses the interaction between SMURF1 and CD47, resulting in decreased CD47 K48‐linked polyubiquitination and degradation.

To identify the ubiquitination sites of CD47 catalyzed by SMURF1, we constructed five singlet mutants (K74R, K85R, K99R, K102R, K290R) of CD47 in MHCC97‐H cells based on the polyubiquitylation prediction website (www.ubpred.org). Among them, K85R, K99R, and K102R mutants showed decreased polyubiquitylation levels as compared to the WT CD47. The triplet mutant (K85/K99/102R) further reduced CD47 polyubiquitylation levels, which were resistant to 2F‐Fuc‐induced upregulation (Figure 3F). Besides, the K85/99/102R mutant abrogated SMURF1‐mediated polyubiquitylation and prolonged CD47 half‐life (Figures 3G; S7E, Supporting Information). Taken together, these data demonstrate that FUT8‐mediated core fucosylation at N111 inhibits SMURF1‐induced CD47 polyubiquitylation at K85/99/102, thereby facilitating CD47 cell surface expression.

### 2.4 Core Fucosylation of CD47 Inhibits Macrophage‐Mediated Tumor Cell Phagocytosis and NK Cell Cytotoxicity

**Figure 4:**
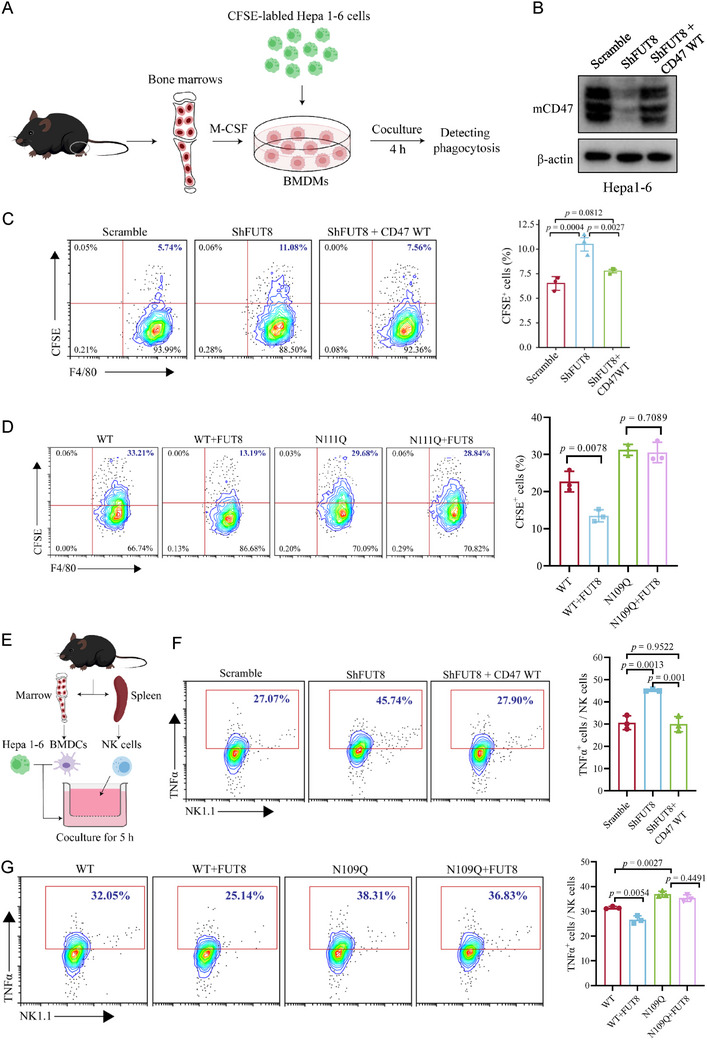
Core fucosylation of CD47 inhibits macrophage‐mediated tumor cell phagocytosis and NK cell cytotoxicity. A) Schematic diagram of phagocytosis analysis in vitro. B) Generation of stable Hepa 1–6 cells with FUT8 knockdown and reconstituted expression of WT CD47. C) Flow cytometry analysis of phagocytosis efficiency of macrophages cultured with Hepa1‐6 cells infected with scramble, shFUT8, and shFUT8 reconstituted with CD47 expression. n = 3; Data are depicted as means ± SD. P‐values were calculated by unpaired two‐tailed Student's t‐tests. D) Flow cytometry analysis of phagocytosis efficiency of macrophages cultured with CD47 WT or N109Q reconstituted Hepa1‐6 cells infected with vector and HA‐FUT8. n = 3; Data are depicted as means ± SD. P‐values were calculated by unpaired two‐tailed Student's t‐tests. E) Schematic diagram of cocultures of CD11c^+^ CD103^+^ BMDCs, Hepa1‐6 cells, and NK cells in Transwell assays (0.4 µm). F) Flow cytometry analysis of TNFα production of NK cells cultured with Hepa1‐6 cells infected with scramble, shFUT8, and shFUT8 reconstituted with CD47 expression. n = 3; Data are depicted as means ± SD. P‐values were calculated by unpaired two‐tailed Student's t‐tests. G) Flow cytometry analysis of TNFα production of NK cells cultured with CD47 WT or N109Q reconstituted Hepa1‐6 cells infected with vector and HA‐FUT8. n = 3; Data are depicted as means ± SD. P‐values were calculated by unpaired two‐tailed Student's t‐tests.

It was reported that CD47 inhibits the phagocytosis of macrophages and the CD103^+^ DC‐mediated recruitment of NK cells in HCC.^[^ ^45^ ^]^ To assess the effects of core fucosylation in regulating CD47 function, we first performed macrophage‐mediated phagocytosis assays. Hepa1‐6 cells were labelled with 5,6‐carboxyfluorescein diacetate succinimidyl ester (CFSE) and co‐cultured with murine bone marrow‐derived macrophages (BMDMs) (**Figure** **4**A). After co‐culturing for 4 h, flow cytometry and immunofluorescence assays were performed to measure CFSE incorporation. The data revealed that the depletion of *FUT8* in Hepa1‐6 cells promoted BMDMs‐mediated phagocytosis (Figure 4B,C). The effect was fully reversed by reconstituted expression of CD47 in *FUT8*‐depleted Hepa1‐6 cells (Figures 4B,C; S8A, Supporting Information). Furthermore, CD47 N109Q reconstituted Hepa1‐6 cells triggered a more powerful phagocytotic response of BMDMs compared to CD47 WT reconstituted cells (Figure S8B,C, Supporting Information). Similar results were observed in CD47 N109A and N109T reconstituted Hepa1‐6 cells (Figure S8D,E, Supporting Information). Overexpression of FUT8 dampened the phagocytosis of CD47 WT reconstituted cells by BMDMs, but had no obvious effect on CD47 N109Q cells (Figures 4D; S8C, Supporting Information). These data suggest that core fucosylation‐mediated CD47 expression repressed tumor cell phagocytosis by macrophages.

To examine the impact of core fucosylation on chemokine‐dependent migration of CD103^+^ DCs, we performed Transwell chemotaxis assays by co‐culturing Hepa1‐6 cells in the bottom chamber and CD103^+^ DCs in the top chamber. The results show that FUT8 knockdown enhances the migration of CD103^+^ DCs and increases expression of chemokine receptor CCR7 on the surface of CD103^+^ DCs (Figure S9A,B, Supporting Information). These effects were rescued by the reconstitution of wild‐type CD47 (Figure S9A,B, Supporting Information). Furthermore, CD47 N109Q‐reconstituted Hepa1‐6 cells induced more DC migration and higher CCR7 expression on DCs than WT reconstituted cells (Figure S9C,D, Supporting Information). FUT8 over‐expression represses WT Hepa1‐6 cells‐induced DCs migration and decreases the CCR7 expression of DCs, while these effects were not observed in N109Q cells (Figure S9C,D, Supporting Information). These results suggest that FUT8‐mediated CD47 core fucosylation inhibits DC migration by downregulating CCR7 expression on DCs.

We next examined the impact of core fucosylation on NK cell cytotoxicity by transwell assays. Hepa1‐6 cells were co‐cultured with BMDCs in the bottom chamber, and NK cells were cultured in the top chamber (Figures 4E; S10A, Supporting Information). After incubation for 5 h, NK cells were collected and subject to flow cytometry analysis. The data showed that *FUT8‐*depleted Hepa1‐6 cells significantly augmented NK cell cytotoxicity, accompanied by elevated levels of TNF‐α and granzyme B (GZMB), the hallmark cytokines released by activated NK cells that mediate tumor cell elimination (Figures 4F; S10B, Supporting Information). Restoration of CD47 expression in *FUT8*‐depleted Hepa1‐6 cells decreased NK cell cytotoxicity to the level observed for control cells (Figures 4F; S10B, Supporting Information). Consistently, NK cells co‐cultured with CD47 N109Q, N109A, and N109T reconstituted Hepa1‐6 cells demonstrated significantly elevated TNF‐α and GZMB expression levels compared to those incubated with CD47 WT reconstituted cells (Figures 4G; S10C,D, Supporting Information). Notably, FUT8 overexpression attenuated NK cell‐mediated cytotoxic activity in CD47 WT reconstituted cells, while exerting negligible influence on CD47 N109Q reconstituted cells (Figure 4G). Collectively, these findings demonstrate that FUT8‐mediated core fucosylation of CD47 suppressed both macrophage‐mediated phagocytosis of tumor cells and NK cell cytotoxic activity.

### 2.5 FUT8‐Mediated Core Fucosylation Promotes Tumor Growth and Tumor Immune Evasion

**Figure 5:**
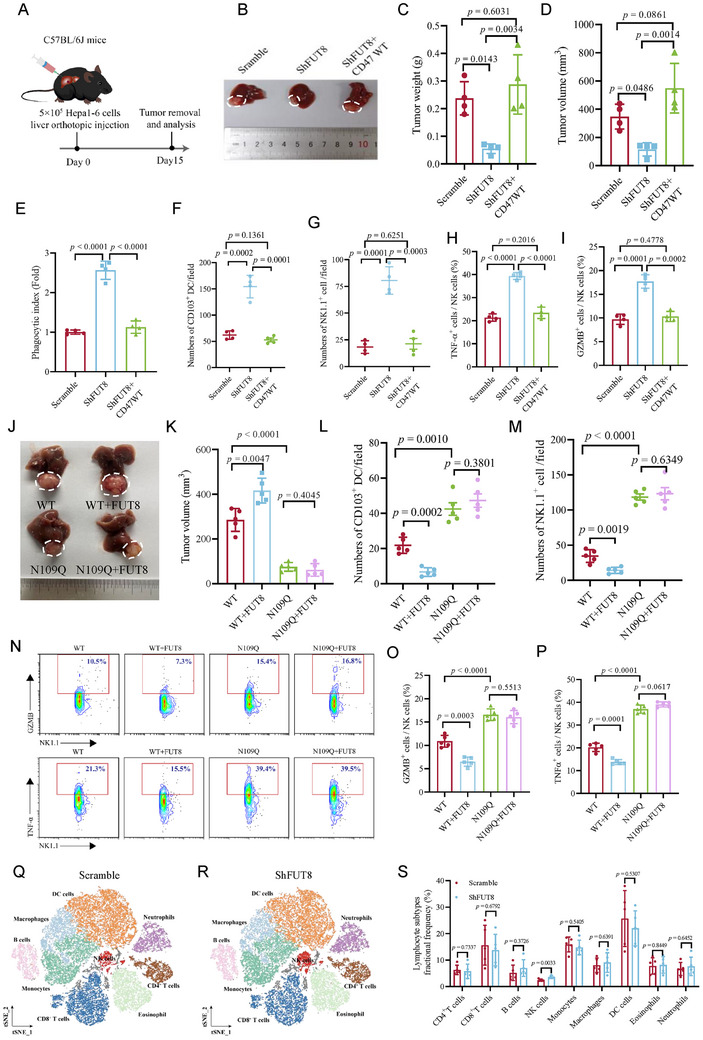
FUT8‐mediated core fucosylation promotes tumor growth and tumor immune evasion. A) Schematic of the orthotopic HCC mouse model. B) Orthotopic tumor formation in C57BL/6J mice from Hepa1‐6 cells infected with scramble, shFUT8, and shFUT8 reconstituted with CD47 expression. C, D) Analysis of tumor weight (C) and volume (D) of tumors from Hepa1‐6 cells infected with scramble, shFUT8, and shFUT8 reconstituted with CD47 expression. n = 4; Data are depicted as means ± SD. P‐values were calculated by unpaired two‐tailed Student's t‐tests. E) Phagocytic index analysis of macrophages in tumors from Hepa1‐6 cells infected with scramble, shFUT8, and shFUT8 reconstituted with CD47 expression. n = 4; Data are depicted as means ± SD. P‐values were calculated by unpaired two‐tailed Student's t‐tests. F, G) Immunohistochemical analysis of tumors from Hepa1‐6 cells infected with scramble, shFUT8, and shFUT8 reconstituted with CD47 expression. Quantification of CD103^+^ DCs (F) and NK1.1^+^ cells (G) infiltration in tumors. n = 4; Data are depicted as means ± SD. P‐values were calculated by unpaired two‐tailed Student's t‐tests. H, I) Flow cytometry analysis of TNFα (H) and GZMB (I) production of NK cells in tumors from Hepa1‐6 cells infected with scramble, shFUT8, and shFUT8 reconstituted with CD47 expression. n = 4; Data are depicted as means ± SD. P‐values were calculated by unpaired two‐tailed Student's t‐tests. J) Orthotopic tumor formation in C57BL/6J mice from CD47 WT or N109Q reconstituted Hepa1‐6 cells infected with vector and HA‐FUT8. K) Analysis of tumor volume weight (L) of tumors from CD47 WT or N109Q reconstituted Hepa1‐6 cells infected with vector and HA‐FUT8. n = 5; Data are depicted as means ± SD. P‐values were calculated by unpaired two‐tailed Student's t‐tests. L, M) Quantification of CD103^+^ DCs (L) and NK1.1^+^ cells (M) infiltration in tumors from CD47 WT or N109Q reconstituted Hepa1‐6 cells infected with vector and HA‐FUT8 by immunohistochemical analysis. n = 5; Data are depicted as means ± SD. P‐values were calculated by unpaired two‐tailed Student's t‐tests. N, P) Flow cytometry analysis of GZMB and TNFα production of NK cells in tumors from CD47 WT or N109Q reconstituted Hepa1‐6 cells infected with vector and HA‐FUT8. n = 5; Data are depicted as means ± SD. P‐values were calculated by unpaired two‐tailed Student's t‐tests. Q, R) Representative t‐SNE map showing the meta‐clustering of CD45^+^ cells in tumors from Hepa1‐6 cells infected with Scramble(Q) and shFUT8 (R). S) Proportion of lymphocyte subtypes in Scramble and shFUT8 groups. n = 5; Data are depicted as means ± SD. P‐values were calculated by unpaired two‐tailed Student's t‐tests.

As CD47 core fucosylation is important in regulating the activity of macrophage and NK cells in vitro, we then investigated its functional impact on tumor growth and immune evasion in vivo. Orthotopic implantation of Hepa1‐6 cells in C57BL/6J mouse livers revealed that *FUT8*‐depleted tumors exhibited significantly reduced tumor volume and weight compared to the scramble control (**Figure** **5**A–D). A similar result was observed in the xenograft model (Figure S11A–C, Supporting Information). Notably, depletion of *FUT8* showed no significant effect on cell proliferation rates in vitro, indicating that *FUT8* depletion repressed tumor growth through immune‐dependent mechanisms (Figure S11D, Supporting Information).

We then performed multiplex immunohistochemistry (mIHC) to quantify the phagocytic clearance of GFP‐labeled tumor cells by macrophages. Quantitative analysis revealed a 2.6‐fold increase in phagocytic index in mice bearing *FUT8*‐depleted Hepa1‐6 cells as compared to the control, suggesting enhanced macrophage‐mediated tumor cell elimination upon *FUT8* depletion (Figures 5E; S11E, Supporting Information). Consistently, IHC analyses of these tumor tissues showed that CD103^+^ DC cell and NK cell infiltration levels were significantly upregulated in *FUT8*‐depleted tumors compared with the control (Figures 5F,G; S11F, Supporting Information). Moreover, the levels of TNF‐α and GZMB in NK cells were significantly increased in *FUT8*‐depleted tumors (Figures 5H,I; S11G, Supporting Information). Reconstituted expression of WT CD47 abrogated these tumor‐inhibitory effects, suggesting that the anti‐tumor effect was dependent on CD47 expression (Figures 5E–I; S11A–G, Supporting Information). Next, we probed the impact of CD47 N109 core fucosylation on tumors in the subcutaneous and orthotopic HCC model. As expected, both CD47 WT and N109Q‐reconstituted Hepa1‐6 cells exhibited comparable proliferation rates in vitro regardless of FUT8 overexpression (Figure S11H, Supporting Information). The tumors generated from CD47 N109Q‐reconstituted cells displayed smaller tumor volumes and masses, which were accompanied by increased macrophage‐mediated tumor cell phagocytosis and infiltration levels of CD103^+^ DC and NK cells, and elevated TNF‐α and GZMB expression in NK cells (Figures 5J–P; S11I–N, Supporting Information). FUT8 overexpression abrogated these effects in the WT tumors, but showed no obvious impact on N109Q tumors (Figures 5J–P; S11I–N, Supporting Information).

To gain a more comprehensive understanding of the tumor immune microenvironment (TIME) upon *FUT8* depletion, we performed the immune cell profiling using mass cytometry by time of flight (CyTOF) with a customized panel targeting key markers across major immune cell types. Dimensionality reduction via t‐SNE revealed distinct immune cell populations within the tumor microenvironment, including CD4^+^ T cells (CD3^+^, CD4^+^), CD8^+^ T cells (CD3^+^, CD8^+^), DCs (MHC‐II^+^, CD11c^+^, CD11b^+^), macrophages (CD11b^+^, F4/80^+^), NK cells (CD3^−^, CD19^−^, NK1.1^+^), monocytes (Ly6C^+^, CD11b^+^), B cells (CD19^+^), eosinophils (Siglec‐F^+^, CD11b^+^) and neutrophils (Ly6G) (Figure 5Q,R). *FUT8*‐depletion tumors exhibited a significantly elevated proportion of NK cells within the CD45^+^ immune cell compartment (Figure 5S). The frequencies of immune cells remained statistically unchanged. Moreover, we observed increased fractions of CD103^+^ DC and CD86^+^ M1‐like macrophages in *FUT8*‐depleted tumors (Figure S12A,B, Supporting Information). Importantly, NK cells in *FUT8*‐depleted tumors displayed enhanced secretion of pro‐inflammatory cytokines, including IFNγ and TNFα (Figure S12C, Supporting Information). However, the CD8^+^ T cell populations, including exhausted CD8^+^ T cells (PD‐1^+^, CD8^+^) or proliferating CD8^+^ T cells (CD8^+^, Ki‐67^+^), remained largely unchanged between *FUT8*‐depleted and control tumors (Figure S12D,E, Supporting Information). These results were consistent with the data that 2F‐Fuc treatment exerts minimal effects on PD‐L1 expression levels and interaction between PD‐L1 and PD‐1 in MHCC97‐H cells (Figure S12F,G, Supporting Information). Thus, FUT8‐mediated core fucosylation modulates the immune landscape by repressing the activity of macrophages and reducing CD103^+^ DC and NK cell abundance. Together, these results suggest that FUT8‐mediated CD47 core fucosylation on N109 promoted tumor growth by repressing the innate immunity.

### 2.6 Blockade of CD47 Core Fucosylation Synergizes with CD47 mAb Therapy

**Figure 6:**
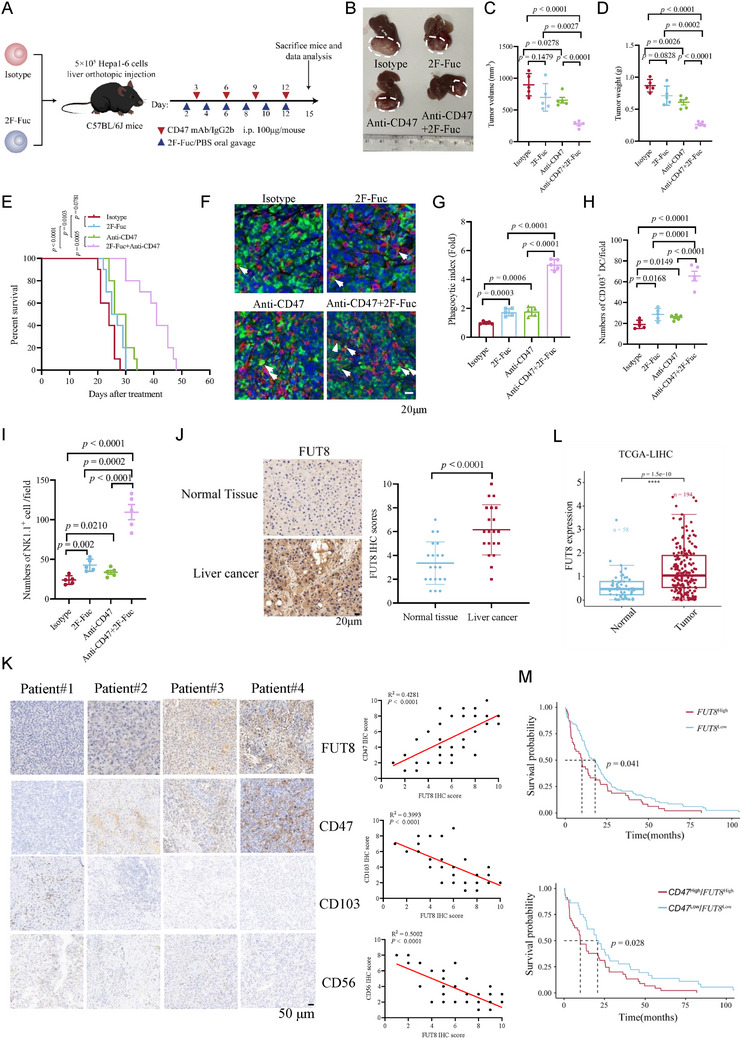
Blockade of CD47 core fucosylation synergizes with CD47 mAb therapy. A) Schematic of synergetic therapy with treatment with 2F‐Fuc treatment and anti‐CD47 antibody in the HCC orthotopic tumor model (n = 5 mice per group). B) Images of tumors harvested from mice bearing Hepa1‐6 cells treated with anti‐CD47 antibody, or 2F‐Fuc, or their combination. C–E) Analysis of tumor volume (C), tumor weight (D), and survival curve of mice (E). n = 5; Data are depicted as means ± SD. P‐values were calculated by unpaired two‐tailed Student's t‐tests. F, G) Multiplex immunohistochemical analysis of tumors (F) and Phagocytic index analysis of macrophages in tumors (G). n = 5; (Scale bar, 20 µm). Data are depicted as means ± SD. P‐values were calculated by unpaired two‐tailed Student's t‐tests. H, I) CD103^+^ DCs (H) and NK1.1^+^ cells (I) infiltration in tumors. n = 5; Data are depicted as means ± SD. P‐values were calculated by unpaired two‐tailed Student's t‐tests. J) Immunohistochemical analysis of FUT8 expression in normal tissues and liver cancer samples. (Scale bar, 20 µm), n = 20; Data are depicted as means ± SD. P‐values were calculated by unpaired two‐tailed Student's t‐tests. K) Analysis of the correlation of FUT8/CD47, FUT8/CD103, and FUT8/CD56 from human HCC samples. (Scale bar, 50 µm), n = 20. L) Analysis of FUT8 mRNA expression in normal and tumor samples from the TCGA database. M) Kaplan–Meier plots of the overall survival of LIHC patients with high and low FUT8 or FUT8/CD47 mRNA expression.

As inhibition of core fucosylation decreased CD47 expression and increased innate immune activity, we speculate that blockade of core fucosylation might boost the effect of CD47 mAb therapy. To test this, we employed a CD47 monoclonal antibody (mAb) to treat C57BL/6 mice inoculated with Hepa1‐6 cells in orthotopic liver with or without 2F‐Fuc treatment (**Figure** **6**A). CD47 mAb treatment modestly reduced tumor volume and weight as compared to the isotype‐treated group (Figure 6B–D). Notably, combined treatment with CD47 mAb and 2F‐Fuc further decreased the tumor volume and weight, and prolonged the survival of mice compared to 2F‐Fuc or CD47 mAb treatment alone, but had no impact on the weight of the mice (Figures 6B–E; S13A, Supporting Information). Additionally, combined treatment enhanced macrophage‐mediated tumor cell phagocytosis and infiltration levels of CD103^+^ DC cells and NK cells (Figures 6F–I; S13B, Supporting Information). To further investigate whether in vivo inhibition of FUT8 could synergize with CD47 mAb therapy, we applied a doxycycline (DOX)‐induced system to deplete *FUT8* (FUT8 iKD) in Hepa1‐6 cells. qPCR analysis and IHC staining confirmed the DOX‐induced *FUT8* depletion in tumors (Figure S13C,D, Supporting Information). As expected, in vivo inducible *FUT8* depletion and CD47 mAb treatment synergistically repressed tumor growth as compared to treatment with DOX or CD47 mAb alone (Figure S13E–H, Supporting Information). To enhance the therapeutic potential of this strategy, we administered oral FUT8 inhibitor (2‐alkynyl‐fucose) combined with anti‐CD47 antibody to tumor‐bearing C57BL/6J mice. In both subcutaneous and orthotopic HCC models, the combination therapy demonstrated significantly greater tumor growth suppression than either 2‐alkynyl‐fucose or CD47 monoclonal antibody alone (Figure S13I–M, Supporting Information). Importantly, mice achieving tumor‐free status following combination therapy remained protected against tumor rechallenge in the subcutaneous HCC model (Figure S13M, Supporting Information). Furthermore, combination treatment increased the frequency of effector memory T cells within tumor‐draining lymph nodes compared to single‐agent regimens, suggesting induction of durable immune memory (Figure S13N, Supporting Information). As CD47 is highly expressed on red blood cells (RBCs), therapeutic targeting of CD47 has been associated with adverse effects such as anemia. Notably, *FUT8* gene expression during erythroid differentiation (RBC maturation) is gradually decreased (Figure S13O,P, Supporting Information).^[^ ^51^, ^52^ ^]^ Furthermore, analysis of single‐cell sequencing datasets demonstrated that FUT8 expression in RBCs is relatively lower compared to cells from other organs (https://www.proteinatlas.org). Collectively, these results suggest that inhibiting core fucosylation of CD47 could potentially address the limitations associated with current monoclonal antibody treatments and represent a safer immunotherapeutic strategy.

Finally, we evaluated the clinical significance of FUT8‐mediated CD47 expression in patients with HCC. Based on the HCC single‐cell database and the Clinical Proteomic Tumor Analysis Consortium (CPTAC), CD47 was positively correlated with FUT8 at both mRNA and protein levels (Figure S14A,B, Supporting Information). We also performed IHC analyses of human HCC tissues, and observed that FUT8 expression levels were upregulated in HCC tissues and positively correlated with CD47 expression levels but inversely correlated with the infiltration of CD103^+^ DC cells and NK cells (Figure 6J,K). These findings were corroborated by analysis of LIHC cohorts based on the TCGA database (Figures 6L; S14C,D, Supporting Information). The Kaplan‐Meier survival analysis also revealed that FUT8 mRNA expression levels were positively correlated with poor survival of patients with HCC. In addition, the survival of FUT8^high^ CD47^high^ patients was remarkably shorter compared to that of FUT8^low^ CD47^low^ patients (Figure 6M). Taken together, these results support a pivotal role of the FUT8‐CD47 axis in immune evasion and tumor progression in HCC.

## 3 Discussion

**Figure 7:**
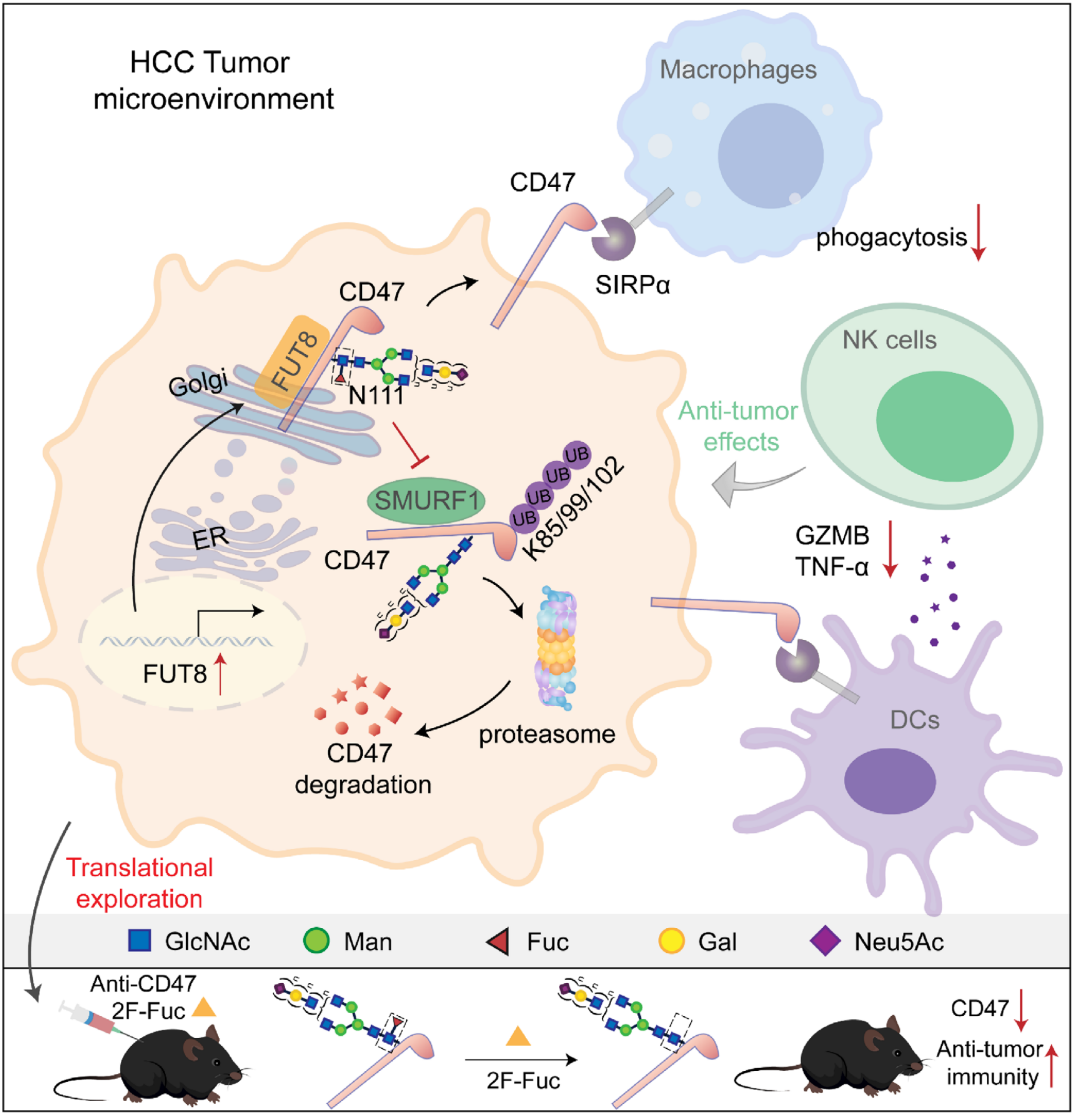
A schematic model showing CD47 core fucosylation regulates innate immune response in HCC.

Tumor cells often upregulate immune checkpoint proteins (ICPs), such as PD‐L1 and CD47, to modulate their interactions with immune cells in order to evade immune detection and destruction.^[^ ^28^ ^]^ How these ICPs are mechanistically regulated in cells is an active area of basic research that also has great potential for translation. ICPs are generally membrane proteins that possess complex forms of glycosylation. Aberrant glycosylation, including truncated O‐glycans, highly sialylated and branched N‐glycans, and hyper‐core fucosylation, is now considered an important hallmark of cancer and critically regulates tumor immunity.^[^ ^53^, ^54^ ^]^ How aberrant glycosylation regulates ICPs is largely unknown. In this study, we demonstrate that FUT8‐mediated core fucosylation augmented CD47 expression to escape innate immune surveillance (**Figure** **7**). Mechanistically, FUT8‐catalyzed core fucosylation of CD47 on N111 inhibited the interaction between CD47 and the E3 ligase SMURF1, thus abrogating SMURF1‐mediated CD47 polyubiquitylation and degradation. Consequently, blockade of CD47 core fucosylation reduced CD47 expression and increased macrophage‐mediated phagocytosis of tumor cells. Moreover, it elevated the infiltration of CD103^+^ DCs, resulting in increased recruitment and activation of NK cells, and consequently inhibited tumor growth in a murine model of HCC. Notably, combined treatment of core fucosylation inhibition and CD47 mAb elicited stronger anti‐tumor effects than either treatment alone in the orthotopic model of HCC. Thus, our findings elucidate a previously uncharacterized mechanism by which glycosylation‐dependent regulation of ICPs modulates the innate immunity in the tumor microenvironment to facilitate tumor growth.

Targeting the CD47‐SIRPα immune checkpoint axis has emerged as a promising therapeutic strategy and is being evaluated in clinical trials in various types of cancer.^[^ ^55^ ^]^ For example, Magrolimab, a mAb that targets CD47, has progressed to Phase III clinical trials for the treatment of myelodysplastic syndromes and acute myeloid leukemia (AML).^[^ ^56^ ^]^ Bispecific antibodies such as TTI‐622, which target CD47 and CD19, along with fusion proteins like ALX148, are under evaluation in Phase I/II trials for their efficacy against lymphoid malignancies and various solid tumors.^[^ ^57^ ^]^ However, the clinical development of CD47‐targeted therapies has been hampered by on‐target hematological toxicities, primarily due to the constitutive expression of CD47 on erythrocyte membranes.^[^ ^58^, ^59^ ^]^ Therefore, optimizing tumor targeting to reduce side effects is a promising way to improve the efficacy of current anti‐CD47 therapies. CD47 is a known glycoprotein with extensive N‐glycosylation. Our study found that CD47 contains high levels of core fucosylation, which is generally considered as tumor‐associated glycans involved in cancer pathogenesis. Our study reveals that FUT8 expression is highly upregulated and positively correlates with CD47 expression levels in HCC. Notably, FUT8 expression is rather low in red blood cells. Thus, targeting both core fucosylation and CD47 might present a more viable and promising approach to overcome the current clinical limitation. As we showed that blockade of core fucosylation downregulated CD47 expression, the combined treatment likely reduced the dosage of anti‐CD47 mAb, with the potential benefit of reducing side‐effects associated with existing clinical mAb therapies.

While our study highlights the critical role of FUT8‐mediated core fucosylation in stabilizing CD47 and promoting tumor immune evasion, quantitative glycoproteomic profiling of FUT8‐overexpressing MHCC97‐H cells revealed a broader landscape of immune‐modulatory proteins regulated by this modification. We identified multiple immune‐related targets, including checkpoint molecules (CD276), cytokine receptors (IL7R, IL1R1, IL6ST, IL18RAP), and trafficking regulators (CD63), that exhibit elevated core fucosylation upon FUT8 overexpression (Figure S14E, Supporting Information). Notably, CD276, a B7 family immune checkpoint linked to poor prognosis in triple‐negative breast cancer (TNBC), has been reported to depend on core fucosylation for its protein stability and immunosuppressive function.^[^ ^37^ ^]^ This suggests a potential synergistic network in which core‐fucosylated CD276 and CD47 may cooperatively dampen anti‐tumor immunity through distinct pathways. Such crosstalk could influence the efficacy of combinatorial therapies targeting these molecules. While the function of core fucosylation on other identified targets requires further validation, our glycoproteomic data underscore FUT8 as a master regulator of immune evasion in HCC, extending beyond CD47 to orchestrate a multifaceted suppressive microenvironment. Future studies dissecting the synergistic effect among these modified targets will refine therapeutic strategies disrupting FUT8‐dependent immunosuppression.

Although our study reveals a previously unknown mechanism for regulating CD47 expression and CD47‐mediated immune evasion, our study still has some limitations. First, we have focused our investigation on HCC. As CD47 and core fucosylation are upregulated in various types of cancer, whether the same glycosylation‐mediated regulation on CD47 and tumor immunity is present in other types of cancer awaits further investigation, which will broaden the scope of CD47‐targeted therapy in cancer. Second, 2F‐Fuc inhibits core fucosylation via depleting the sugar donor GDP‐Fuc pool, rendering it not a selective inhibitor of core fucosylation. Development of selective inhibitors of FUT8 will undoubtedly pave the way for designing more effective CD47‐mediated cancer immunotherapies.

## 4 Experimental Section

### Cell Culture and Tumor Tissues

MHCC97‐H, HCCLM3, and Hepa1‐6 cells were obtained from American Type Cell Culture (ATCC). All cell lines were cultured in Dulbecco's Modified Eagle's Medium supplemented with 10% fetal bovine serum (FBS) and 1% penicillin–streptomycin at 37 °C under 5% CO_2_. The cell lines were tested, and no mycoplasma was found. Bone marrow‐derived macrophages (BMDMs) isolated from the femurs and tibiae of C57BL/6J mice were cultured in Iscove's Modified Dulbecco's Medium (Gibco) containing 10% FBS and supplemented with murine M‐CSF (20 ng mL^−1^, CK02, Novoprotein) for 7 days, and were digested for further use. Bone marrow‐derived dendritic cells (BMDCs) also isolated from the femurs and tibiae of C57BL/6J mice were cultured in Iscove's Modified Dulbecco's Medium (Gibco) containing 10% FBS and supplemented with murine GM‐CSF (25 ng mL^−1^, 315‐03, PeproTech) and murine FLT3LG (160 ng mL^−1^, CK 93, Novoprotein). The BMDCs were cultured in bacterial Petri dishes for 10 days and isolated using a mouse CD11c positive selection kit (#18780A, STEMCELL) for co‐culture experiments. A mouse NK Cell Isolation Kit (#19855, STEMCELL) was used to purify mouse NK cells from the spleen of C57BL/6J mice. NK cells were cultured in complete RPIM‐1640 medium (Gibco) supplemented with murine IL‐2 (25 ng mL^−1^, 212‐12, PeproTech) for further use. Human hepatocellular carcinoma and peritumoral tissues were obtained from patients at the First Affiliated Hospital of Zhejiang University (Hangzhou, China).

The protocols involving human participants were sanctioned by the Ethical Committee of the School of Medicine at Zhejiang University.

All other experimental methods are provided in the Supporting Information Appendix.

### Data, Materials, and Software Availability

The mRNA expression data were downloaded from the TCGA database and the Genotype‐Tissue Expression (GTEx) project. Raw gene expression counts of HCC samples and normal liver samples were analyzed in R (version 4.2.1). DESeq2 (version 1.36.0) was used to calculate the fold change and *p*‐value of FUT8 relative expression in HCC samples compared to normal liver samples. Normalized protein expression data of 171 TCGA liver cancer samples (PDC000198) were downloaded from the Clinical Proteomic Tumor Analysis Consortium (CPTAC). The correlation of FUT8 gene expression and activated NK cell infiltration was performed in the R package CIBERSORT (version 0.1.0).

## Conflict of Interest

The authors declare no conflict of interest.

## Author Contributions

Y.C. and S.C. contributed equally to this work. Q.Z. and W.Y. conceived the project and designed cell biology experiments. Y.C., M.L., J.H., and X.C. performed cell biology and biochemistry experiments. B.L. and L.W. provided tumor tissue samples. Q.Z. and W.Y. analyzed the data and wrote the paper with inputs from all authors.

## Supporting information



Supporting Information

## Data Availability

The data that support the findings of this study are available from the corresponding author upon reasonable request.
