# Supplementary material for: Core Fucosylation Represses SMURF1‐Dependent Degradation of CD47 to Promote Tumor Immune Evasion
Source: Adv Sci (Weinh). 2025 Dec 7;13(11):e16863. doi: 10.1002/advs.202516863 (PMC12931233; doi:10.1002/advs.202516863)
Supplement: Supplementary file 1 — Supporting Information [file ADVS-13-e16863-s001.pdf]

METHOD

Cell culture and tumor tissues

MHCC97-H, HCCLM3 and 293T cells were obtained from American Type Cell Culture (ATCC). Hepa1-6 cells were obtained from Ubigene Biosciences (YC-A005, Guangzhou, China). All cell lines were cultured in Dulbecco's Modified Eagle's Medium supplemented with 10% fetal bovine serum (FBS) and 1% penicillin-streptomycin at 37 °C under 5% CO_2. The cell lines were tested and no mycoplasma was found. Bone marrow-derived macrophages (BMDMs) isolated from the femurs and tibiae of C57BL/6J mice were cultured in Iscove's Modified Dulbecco's Medium (Gibco) containg 10% FBS and supplemented with murine M-CSF (20 ng/ml, CK02, novoprotein) for 7 days, and were digested for further use. Bone marrow-derived dendritic cells (BMDCs) also isolated from the femurs and tibiae of C57BL/6J mice were cultured in Iscove's Modified Dulbecco's Medium containg 10% FBS and supplemented with murine GM-CSF (25 ng/ml, 315-03, PeproTech) and murine IL4 (20 ng/ml, CK15, novoprotein). The BMDCs were culture for 10 days and isolated using a mouse CD11c positive selection kit (#18780A, STEMCELL) for co-culture experiments. A mouse NK Cell Isolation Kit (#19855, STEMCELL) was used to purify mouse NK cells from the spleen of C57BL/6J mice. NK cells were cultured in complete RPIM-1640 medium (Gibco) supplemented with murine IL-2 (25 ng/ml, 212-12, PeproTech) for further use. Human hepatocellular carcinoma and peritumoral tissues were obtained from patients at the first Affiliated Hospital of Zhejiang University (Hangzhou, China). This study was approved by the Ethics Committee of Zhejiang University School of Medicine, with all participants providing informed consent before participation.

Construction of plasmids and stable cell lines

For CD47, FUT8, SMURF1 knockdown, corresponding small hairpin RNAs (shRNAs) were cloned to pLKO.1-EGFP-PURO plasmid. HEK293T cells were used to produce lentivirus. MHCC97-H, HCCLM3 and Hepa1-6 cells were infected with

lentivirus and selected with 2 μg/ml puromycin for 2 weeks to construct stable cell lines. For constructing CD47 reconstituted cell lines, shRNA targeting the 3' UTR sequence of CD47 gene and Flag-tagged WT or N111Q/N109Q CD47 sequence were cloned to pLenti-FlagN-shRNA vector respectively. MHCC97-H, HCCLM3 and Hepa1-6 cells were infected with lentivirus and selected with 2 μg/ml puromycin for 2 weeks. For FUT8, SMURF1 overexpression, HA or Myc-tagged corresponding sequences were cloned in pLVX-HA-IRES-Puro vector. MHCC97-H, HCCLM3 and Hepa1-6 cells were infected with concentrated lentivirus and selected with 2 μg/ml puromycin for 2 weeks. Primer sequences for ShRNA are provided in Supplementary Table 2.

Cell proliferation assay

Cell proliferation ability was measured by Cell Counting Kit-8 (Beyotime, C0039). 1×10^3 cells were placed into a 96-well plate per well. CCK-8 solution was added followed by incubation for 1 h at 37 °C. Absorbance was detected at 450 nm using the Multi-Mode Plate Reader (BioTek).

Immunoprecipitation and immunoblotting analysis

Cells were lysed in RIPA buffer (Beyotime, P0013C) containing a protease inhibitor cocktail (MedChemExpress, HY-K0010). To immunoprecipitate exogenous proteins, Anti-Flag-M2 beads were used to enrich flag-tagged proteins. To immunoprecipitate endogenous proteins, Protein A/G beads were used to incubate with cell lysis and CD47 antibody or IgG. After incubation for 8h, the beads were washed with ice-cold H150 buffer (50 mM Tris HCl, pH 7.4, 150 mM NaCl, 0.5% NP-40, 10% Glycerol). 3X Flag peptides in TBS (Beyotime, P9801) were used to elute the bounding proteins. Equal aliquots of protein were loaded on 8% or 10% SDS-PAGE gel and then transferred to 0.45 μm PVDF membranes (Millipore, IPVH00010). After blocking with 5% non-fat milk, the membrane was incubated with primary corresponding antibodies at 4 °C overnight. The membrane was washed with 1X PBST 3 times for

10 minutes each and incubated with secondary antibodies. The information of indicated antibodies is provided in Supplementary Table 1.

Immunofluorescence and Immunohistochemistry staining

Immunofluorescence staining was performed following standardized protocols. Briefly, cells were cultured on sterile glass coverslips for 24 h, then fixed with 4% (w/v) paraformaldehyde (PFA) solution for 20 min at room temperature (RT). After fixation, cells were washed three times with PBS and permeabilized with 0.2% Triton X-100 for 15 min. After incubation with 3% BSA for 1 h at RT, the cells then were incubated with primary antibody at 4 °C overnight. After washing three times with 1× PBST, anti-mouse or rabbit Alexa Fluor 488 or 594 dye was added and incubated for 1 h. Finally, nuclei were stained with DAPI for 15 min. The images were captured on an FV3000 confocal microscope (Olympus).

For immunohistochemistry analysis, following fixation, the tissues were fixed in 4% paraformaldehyde and embedded through paraffin and sectioned at 6 μm thickness. Tissue sections were subjected to deparaffinization with xylene followed by rehydration through a graded ethanol series (100%-70%). Heat-induced antigen retrieval was carried out in citrate buffer (pH 6.0) or ethylenediaminetetraacetic acid buffer (pH 9.0) using a 95 °C water bath for 40 min. Immunohistochemical staining was conducted with the VECTASTAIN Elite ABC-HRP Kit (Vector Laboratories, Burlingame, CA) in accordance with the manufacturer's protocol. The sections were stained by anti-CD103 and anti-NK1.1 antibody. Sections of paraffin-embedded human HCC samples were stained with indicated antibodies. Tissue sections were semi-quantitatively assessed using a composite scoring system incorporating both staining intensity and distribution. Intensity was graded as: 0 (negative), 1-2 (weak), 3-4(moderate), or 5-6 (strong). The percentage of positive cells was categorized as: 1 (<33%), 2 (33-66%), or 3 (>66%). The final histoscore (range 0-9) was calculated by multiplying the intensity and distribution scores.

In vitro phagocytosis assays

BMDMs were prepared as described above. 2×10^6 indicated Hepa1-6 cells were labeled with CFSE using the CellTrace CFSE Cell Proliferation Kit (Thermo Fisher Scientific), and cultured with 5×10^5 BMDMs at 37 °C for 4 h in 6-well plates. BMDMs were starved for 2 h before co-cultured with Hepa1-6 cells. Cells were harvested and stained with APC anti-mouse F4/80 Antibody (Biolegend, 123116). Flow cytometry was performed to analyze the CFSE intensity in F4/80$^+$ cells. For visualizing phagocytosis by immunofluorescence, 2×10^5 indicated Hepa1-6 cells were cultured with 5×10^4 BMDMs at 37 °C for 4 h in 24-well plates inserted with corresponding glass slides. Then the cells were fixed and stained with F4/80 antibody (abcam, ab300421). Images were captured by a confocal laser scanning microscope (Olympus FV3000).

Determination of CD47 half-life

Cycloheximide (CHX; MedChemExpress, HY-12320) was employed at 100 μM to inhibit new protein synthesis. At predetermined time points post-CHX treatment, cells were harvested and lysed. CD47 protein stability was assessed by western blot analysis. The band intensity was quantified to determine relative half-life using Image J.

Glycosylation analysis of CD47 in vitro

To analyze CD47 glycosylation patterns, cell lysates or immunoprecipitated CD47 protein were treated with PNGase F (New England BioLabs, P0704S) and O-glycosidase (New England BioLabs, P0733) following the manufacturer's protocol. Briefly, samples were incubated with recombinant glycosidases in the provided buffer at 37°C for 4 h. Reactions were terminated by adding 4× SDS sample loading buffer, and CD47 protein levels were assessed by immunoblotting using the indicated antibody.

Glycosylation analysis of CD47 in live cells

Cells were pre-seeded in six-well plates and treated with dimethyl sulfoxide (DMSO), Tunicamycin (2 μg/ml, MedChemExpress, HY-A0098), Thiamet G (10 μM, MedChemExpress, HY-12588) or PUGNAc (100 μM, MedChemExpress, HY-108241) for 24 h. Cells were harvested and lysed for immunoblotting analysis.

PD-1 and PD-L1 interaction assay

To measure PD-1 and PD-L1 proteins interaction, MHCC97-H cells were fixed in 4% paraformaldehyde at room temperature for 15 minutes and then incubated them with recombinant human PD-1 Fc protein (R&D Systems) for 1 hour at room temperature. Wash cells with PBS twice. The secondary antibodies used were anti-human Alexa Fluor 488 dye conjugate (Thermo Fisher Scientific). Wash cells with PBS twice. The binding intensity was analyzed by flow cytometry.

HCC orthotopic xenograft model in mice

All procedures involving animals were reviewed and approved by the Institutional Animal Care and Use Committee (IACUC) of Zhejiang University (ZJU20250414), and were conducted in strict compliance with the NIH Guide for the Care and Use of Laboratory Animals. A total of 5×10^5 indicated Hepa1-6 cells suspended in 100 μL PBS:Matrigel (1:1, v/v) mixture and were orthotopically inoculated into the hepatic capsule of 6-week-old male C57BL/6J mice (n = 5). For combinational antitumor efficacy experiments, GFP-expressing Hepa 1-6 cells were pre-treated with 200 μM 2F-Fuc or DMSO for a week before inoculation. The anti-CD47 mAb (BioXCell) was delivered via i.p. injection at 100 μg/per mouse at the indicated time points. 2F-Fuc was given by oral gavage as 3.51 mg/ml in PBS at the indicated time points. 2-alkynyl-fucose (1mg/ml) in water was given during the period of therapy. Doxycycline (Dox, MedChemExpress, HY-N0565) was administered via drinking water at a concentration of 1 mg/mL. The mice were euthanized before tumors volume reached 2 cm^3. Tumor volumes were calculated by the formula: $0.5 \times \text{length} \times \text{width}^2$ in millimeters. Part of the tumors were subjected to flow cytometry analysis, and the other part was used for histochemical analysis.

HCC xenograft model in mice

A total of 5×10^5 indicated Hepa1-6 cells suspended in 100 μL PBS and were subcutaneously inoculated in 6-week-old male C57BL/6J mice (n = 5). When more than 50% of mice in the combination-treatment group achieve complete tumor regression, re-challenge mice with 5×10^5 Hepa1-6 cells.

RPLC-MS/MS for Glycan Profiling of CD47

CD47 proteins isolated from LM3 cells were resuspended and denatured using 30 μL of 8 M urea, followed by dilution with 50 mM ammonium bicarbonate (ABC) solution. The proteins were treated with 200 mM tris (2-carboxyethyl) phosphine (TCEP) at 55°C for 1 h, followed by alkylation with 200 mM iodoacetamide (IAA) in the dark at RT for 30 min. The reduced and alkylated proteins were further diluted to 2 mL with 50 mM ABC and digested overnight at 37°C using 7 μg of trypsin (enzyme-to-protein ratio of 1:50 w/w). The resulting peptides were dried in a SpeedVac and reconstituted in 500 μL of 0.1% trifluoroacetic acid (TFA). The peptides were desalted using a C18 solid-phase extraction (SPE) column (Phenomenex, 15 μm, 300 Å), which was first activated with 80% acetonitrile (ACN) and equilibrated with 0.1% TFA. After loading the peptide solution, the column was washed with 0.1% TFA, and peptides were eluted sequentially with 50% ACN/0.1% TFA and 80% ACN/0.1% TFA. Separation was performed on a Dionex Ultimate 3000 RSLC nano-HPLC system using a 70 cm analytical column (75 μm inner diameter) packed with C18 particles (300 Å, 5 μm, Phenomenex). Mobile phases consisted of 0.1% formic acid (FA) in water (Buffer A) and 0.1% FA in ACN (Buffer B). The peptides were ionized using electrospray ionization (ESI) at 1.9 kV and analyzed on a Q Exactive mass spectrometer (Thermo Fisher Scientific). Full MS scans were acquired in the 700–2000 m/z range at a resolution of 60,000, followed by data-dependent MS/MS scans (Top 20) with higher-energy collisional dissociation (HCD) at a resolution of 30,000. Key parameters included an automatic gain control (AGC) target of 3×10^6 for MS and 5×10^5 for MS/MS, maximum injection times of 20

ms (MS) and 250 ms (MS/MS), an isolation window of 1.4 m/z, and dynamic exclusion for 30 s. Stepped collision energies were set to 20%, 30%, and 31%, and the ion transfer capillary temperature was maintained at 300°C.

Coculture with Transwell assay in vitro

2×10^6 indicated Hepa1-6 cells and 2×10^6 BMDCs were co-cultured in the bottom chamber, and 3×10^5 NK cells were cultured in the upper chamber with a 0.4-μm pore polyester membrane insert for 5 h (CLS3460-48EA, Corning). NK cells were harvested and cell-killing profile was analyzed by Flow Cytometry.

Transwell chemotaxis assay in vitro

Migration assays were performed in the 24-well plates inserted with 8 μm pore size transwell filters (JET, TCS020024). 4×10^5 indicated Hepa1-6 cells were cultured in the bottom chamber, and 1×10^5 BMDCs were cultured in the upper chamber for 24h. Invaded cells on the underside of the membrane were fixed in 4% paraformaldehyde (Beyotime, P0099) and stained with crystal violet solution (Beyotime, C0121). For flow cytometry to examine CCR7 expression, 3×10^6 indicated Hepa1-6 cells were cultured in the bottom chamber, and 5×10^5 BMDCs were cultured in the upper chamber with a 0.4-μm pore polyester membrane insert for 24h. BMDCs were harvested and CCR7 expression was analyzed by Flow Cytometry.

RNA extraction and quantitative Real-Time PCR Analysis

Total RNA was extracted using traditional Trizol method. Genome DNA removement and reverse transcription reaction was performed using HiScript III RT SuperMix for qPCR (Vazyme, R323). The real-time PCR analysis was executed by CFX96™ Real-Time System (BIO-RAD).

Flow Cytometry Staining

For experiments in vitro, cells were harvested and washed twice with PBS. BMDMs were stained with anti-mouse F4/80 APC (123115). BMDCs were stained with

anti-mouse CD11c FITC (117305), anti-mouse CD103 APC (121413), and anti-mouse CCR7 Brilliant Violet 421™ (120119). NK cells were stained anti-mouse NK1.1 PE (108707). For experiments in vivo, mice were sacrificed at the designated time points and tumor tissues were surgically excised. The resected tissues were mechanically dissociated and enzymatically digested using Dispase II (YEASEN, 40104ES60), recombinant DNaseI (Takara, 2270A), and Collagenase Type IV (Gibco, 17104019) to generate single-cell suspensions. Mononuclear cells were isolated using Percoll (YEASEN, 40501ES60) by density gradient centrifugation. For cell surface protein staining, BD Pharmingen™ APC-Cy™7 rat anti-mouse CD45 (557659) and anti-mouse NK1.1 PE (108707) were used. For intracellular cytokine staining, 1 µl BD GolgiPlug™ Protein Transport Inhibitor (555029, BD Pharmingen, San Diego, CA) were used for every 1 mL of cell culture (10^6 cells/mL) and mix thoroughly. Cells were cultured at 37 °C for 6 h. Then BD Cytofix/Cytoperm was used for fixation and permeabilization. Anti-mouse Gramzyme B Brilliant Violet 421™ (396413) and anti-mouse TNFα APC (506307) were used to perform intracellular labelling. For the analysis of effector memory T cells ($CD8^+$ $CD44^+$ $CD62L^-$) in the tumor-draining lymph nodes, BD Pharmingen™ APC-Cy™7 rat anti-mouse CD45 (557659), anti-mouse CD8a PE/Cyanine7 (100722), anti-mouse/human CD44 APC (103011), and anti-mouse CD62L FITC (104405) were used.

CyTOF analysis

Tumor samples were retrieved from storage solution and rinsed twice with cell culture medium. Following this, tissues were finely minced into approximately 1 mm^3 pieces before being enzymatically dissociated in a digestive enzyme mixture diluted in medium (final volume: 5 mL). This digestion was performed in a shaking incubator at 37°C for one hour. The resulting cell suspension was filtered through a 70 µm strainer, and the cells were pelleted by centrifugation at 300 g for 5 minutes at 4°C. The cell pellet was resuspended in Cell Staining Buffer (CSB) and counted. For CyTOF analysis, cells were first stained with cisplatin-194Pt for live/dead discrimination (5 minutes), followed by blocking of Fc receptors (20 minutes). An antibody cocktail

was then used to stain surface markers (30 minutes). Intracellular or nuclear markers were subsequently stained using specific antibodies (30 minutes). Cells were stained with a metal-tagged antibody cocktail (30 minutes), resuspended in deionized water, and spiked with EQ Four Element Calibration Beads before acquisition on a calibrated CyTOF instrument. Acquired data underwent debarcoding using a doublet-filtering method, normalization across batches with bead normalization, and pre-gating (FlowJo) to eliminate debris, dead cells, and doublets. Data analysis was conducted via X-shift clustering, and cell populations were identified based on marker expression heatmaps. t-SNE dimensional reduction was utilized to visualize population distributions and marker expression.

Multiplex immunohistochemical (mIHC)

Multiplex immunohistochemistry was performed following the manufacturer's instructions (abs50012). Paraffin-embedded mouse tumor tissue slides were first hydrated through sequential washes in xylene and anhydrous ethanol. After hydration, antigen retrieval was conducted by incubating the slides in preheated retrieval buffer (pH 9.0 EDTA or pH 6.0 citrate buffer) at 100°C for 25 min. The slides were then cooled to RT and blocked with peroxidase to minimize nonspecific binding. For antibody staining, slides were incubated with the primary antibody at RT for 1 h or at 4°C overnight, followed by incubation with the corresponding secondary antibody (anti-mouse or anti-rabbit) conjugated to a fluorescent dye. To enable sequential multiplex staining, the entire process, including antigen retrieval, blocking, and antibody incubation, was repeated for each additional antibody and fluorescent dye combination. After completing all staining cycles, nuclei were counterstained with DAPI, and slides were coverslipped for imaging.

Analyses of TCGA data

The mRNA expression data was downloaded from TCGA database and the Genotype-Tissue Expression (GTEx) project. Raw gene expression counts of HCC samples and normal liver samples were analyzed in R (version 4.2.1). DESeq2

(version 1.36.0) was used to calculate the fold change and *p* value of FUT8 relative expression in HCC samples compare to normal liver samples. Normalized protein expression data of 171 TCGA liver cancer samples (PDC000198) were downloaded from the Clinical Proteomic Tumor Analysis Consortium (CPTAC). The correlation of FUT8 gene expression and activated NK cell infiltration was performed in R package CIBERSORT (version 0.1.0).

Statistics

Quantitative data are expressed as mean ± SD from ≥3 independent experiments. Intergroup comparisons were analyzed by Student's unpaired two-tailed t-test, with $p < 0.05$ defining statistical significance (95% confidence interval).

Data availability

All data supporting the findings of this study are available from the corresponding author on reasonable request.

Supplementary Figures

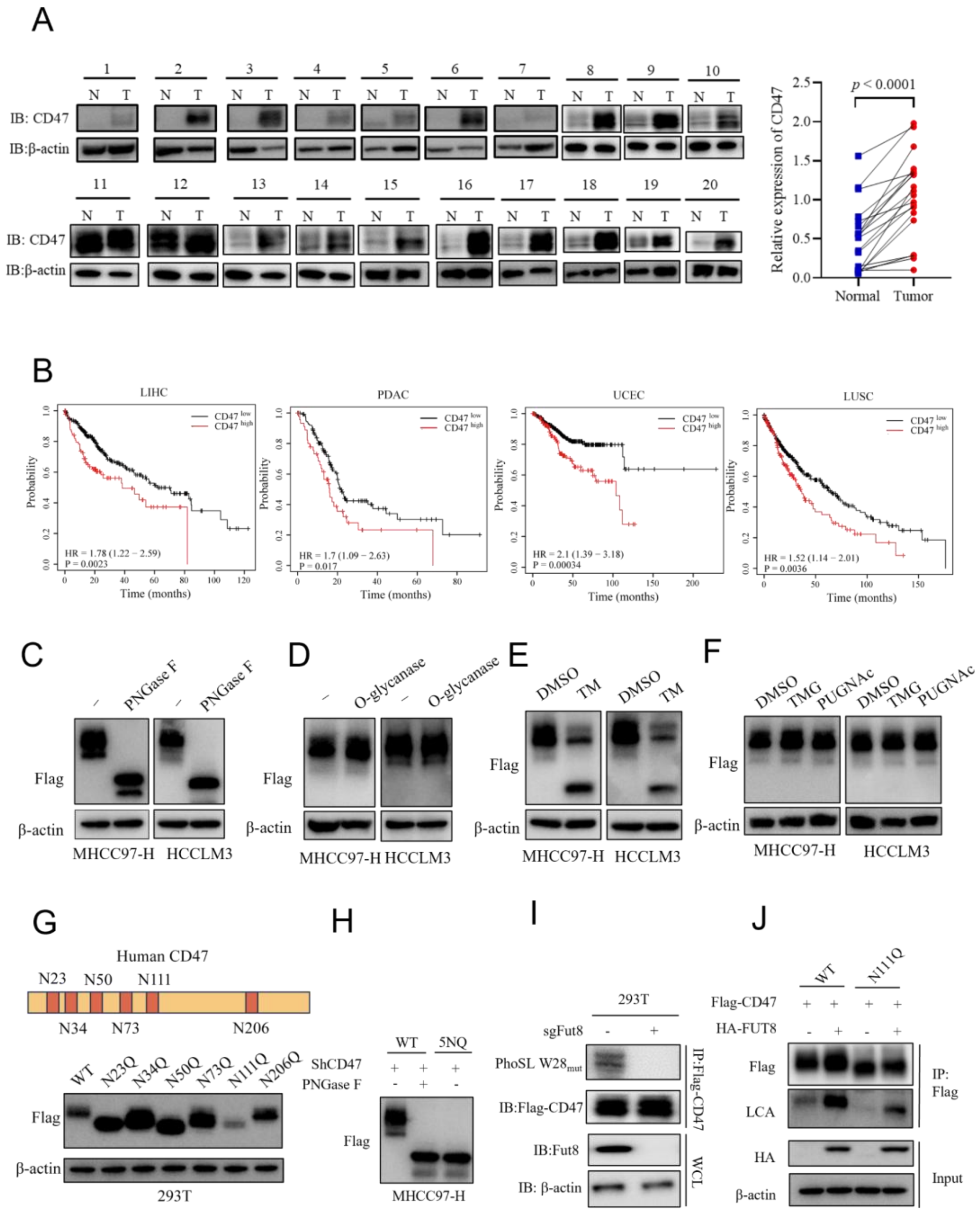

Fig. S1. A, Expression of CD47 protein in 20 representative human HCC fresh samples by immunoblot. Data was quantified. n = 20; Data are presented as means ± SD. **B,** Kaplan–Meier plots of the overall survival of patients with LIHC, PDAC, UCEC, LUSC, stratified by protein expression of CD47. **C,** Immunoblotting analysis of CD47 WT treated without and with PNGase F in MHCC97-H and HCCLM3 cells. **D,** Immunoblotting analysis of CD47 protein expression treated without and with O-glycanase in MHCC97-H and HCCLM3 cells. **E,** Immunoblotting analysis of

CD47 protein expression in MHCC97-H and HCCLM3 cells treated without and with TM (4ug/ml). **F,** Immunoblotting analysis of CD47 protein expression in MHCC97-H and HCCLM3 cells treated without and with TMG (50μM), PUGNAc (100μM). **G,** Six potential N-glycosylated sites of human CD47 and the immunoblotting analysis of CD47 WT and mutants. **H,** Immunoblotting analysis of non-glycosylated CD47 and CD47 WT protein expression upon PNGase F treartment. **I,** Lectin blotting of WT or N111Q CD47 with LCA in 293T cells expressing vector and HA-FUT8.

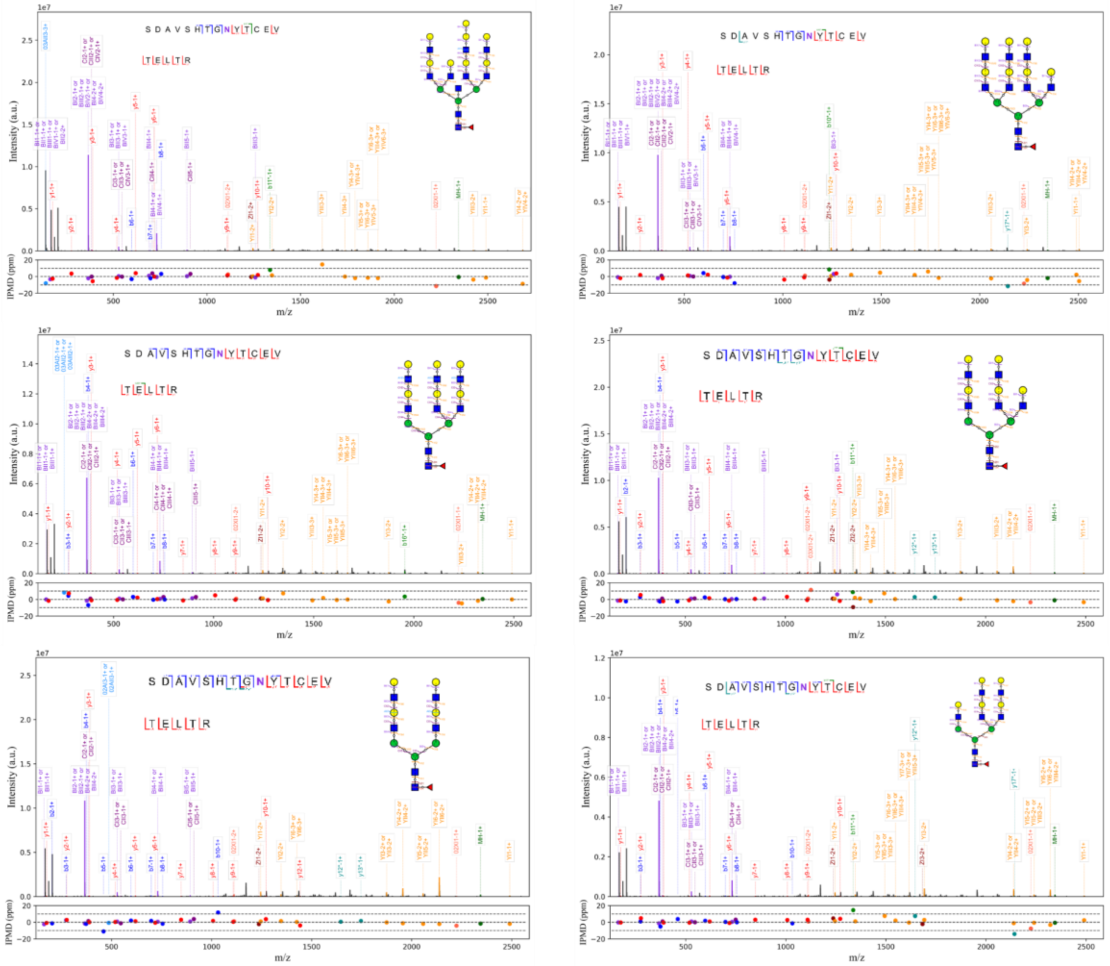

Fig. S2. RPLC-MS/MS of the top six N-glycans on N111 site of purified human CD47 protein.

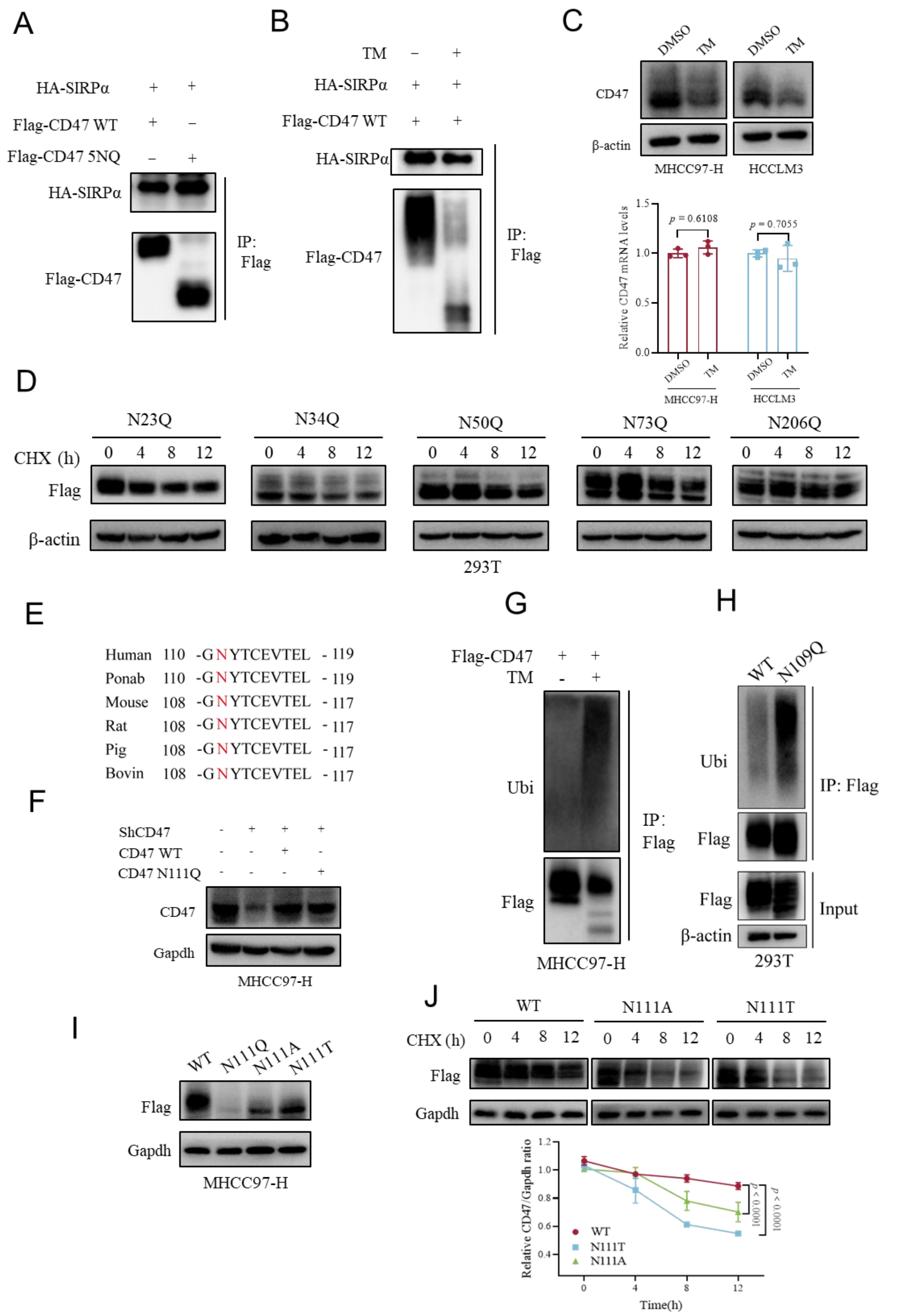

Fig.S3. A, Interaction between CD47 WT or 5NQ mutant and SIRPα by Western blot. **B,** Interaction between CD47 WT and SIRPα in the absence and presence of TM (1ug/ml) by Western blot. **C,** Immunoblotting of CD47 expression and RT-qPCR analysis of CD47 mRNA level in MHCC97-H and HCCLM3 cells in the presence or

absence of TM (4ug/ml) treatment. Data was quantified. n = 3; Data are presented as means ± SD. P values were determined by unpaired two-tailed Student's t tests. **D,** Immunoblotting of CD47 protein in 293T cells expressing N23Q. N34Q, N50Q, N73Q, N206Q CD47 by CHX treatment. **E,** Evolutionary conservation of the N111 residue across species. **F,** Generation of stable MHCC97-H cells with CD47 knockdown and reconstituted expression of shRNA-resistant WT or N111Q CD47. The CD47 knockdown efficiency and re-expression were examined by immunoblotting. **G,** Ubiquitination level of CD47 in MHCC97-H cells upon TM treatment. **H,** Ubiquitination level of CD47 in 293T cells expressing mouse WT or N109Q CD47. **I,** WT, N111Q, N111A, and N111T CD47 expression in endogenous CD47-depleted MHCC97-H cells. **J,** Immunoblotting of CD47 protein in MHCC97-H cells expressing WT, N111A. N111T CD47 by CHX treatment. Data was quantified. n = 3; Data are presented as means ± SD. P values were determined by unpaired two-tailed Student's t tests.

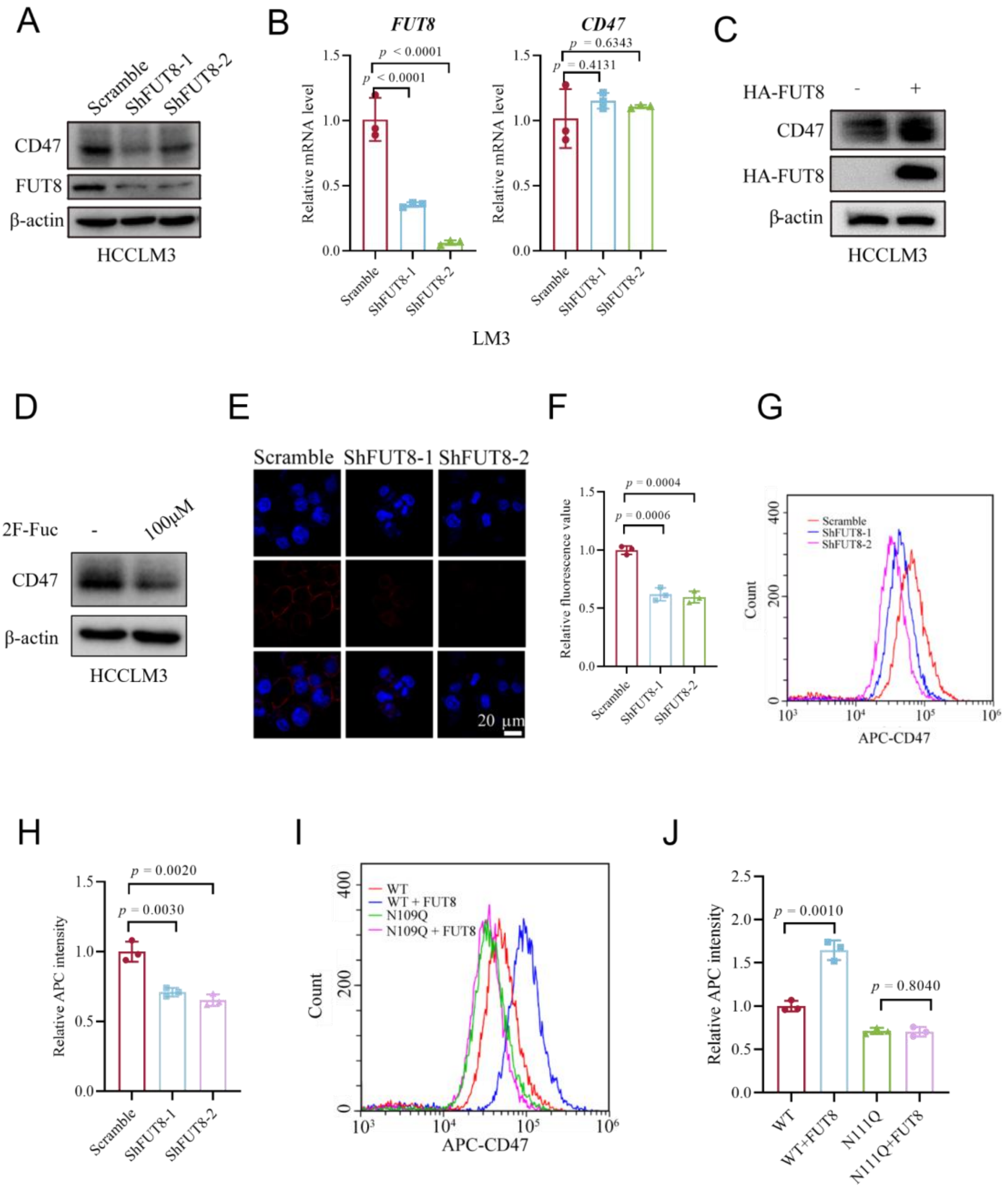

Fig. S4. A, Immunoblotting of CD47 protein in HCCLM3 cells expressing sramble or shFUT8. **B,** Quantitative PCR analysis of FUT8 and CD47 mRNA levels in HCCLM3 cells expressing sramble or shFUT8. n = 3; Data are presented as means ± SD. P values were determined by unpaired two-tailed Student's t tests. **C,** Immunoblotting of CD47 protein in HCCLM3 cells expressing control vector or HA-FUT8. **D,** Immunoblotting of CD47 protein in HCCLM3 cells treated with DMSO or 2F-Fuc (100μM). **E and F,** Immunofluorescence measuring CD47 protein on the cell membrane in MHCC97-H cells expressing scramble and shFUT8. (Scale bar, 20 μm.) (E). Relative fluorescence value was quantified (F). n = 3; Data are

presented as means ± SD. P values were determined by unpaired two-tailed Student's t tests. **G and H,** Flow cytometry measuring CD47 protein on the cell membrane in MHCC97-H cells expressing scramble and shFUT8. (Scale bar, 20 μm.) (G). Median fluorescence intensity (MFI) was measured (H). n = 3; Data are presented as means ± SD. P values were determined by unpaired two-tailed Student's t tests. **I and J,** Flow cytometry analysis of CD47 protein expression on the cell membrane in CD47 WT or N111Q reconstituted MHCC97-H cells expressing control vector or HA-FUT8 (I). Median fluorescence intensity (MFI) was measured (J). n = 3; Data are depicted as means ± SD. P-values were calculated by unpaired two-tailed Student's t-tests.

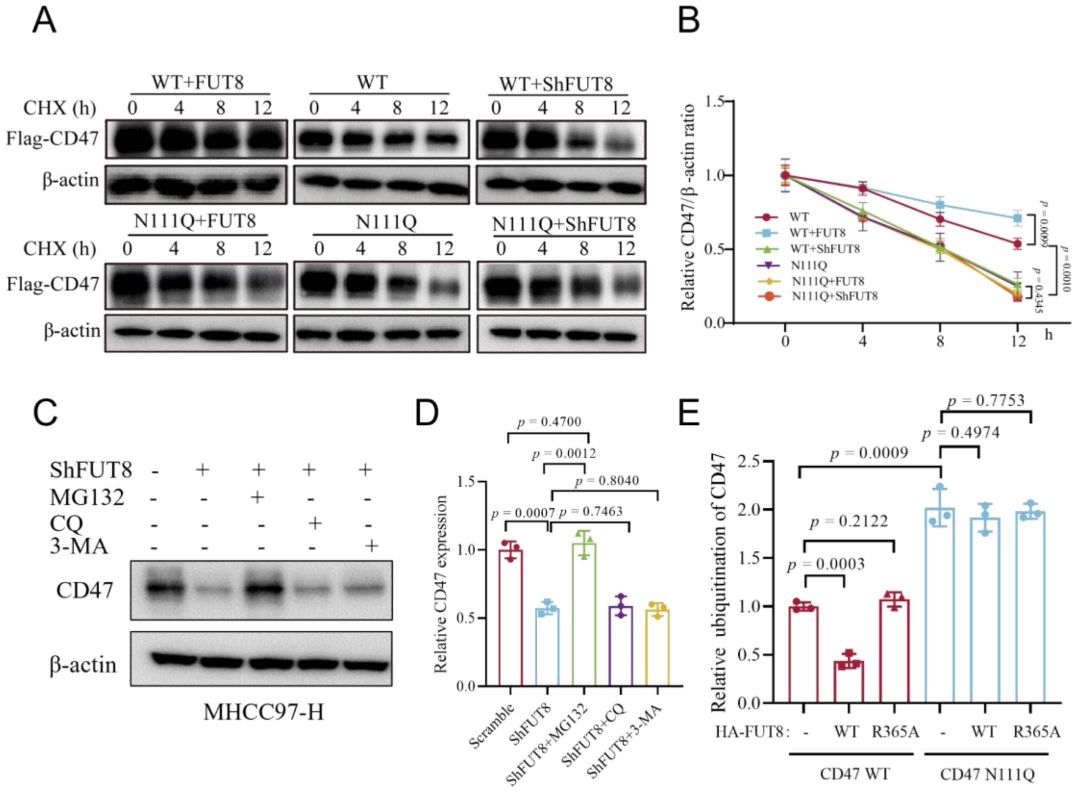

Fig. S5. A and B, Immunoblotting of CD47 WT and N111Q levels in MHCC97-H cells expressing HA-FUT8 or shFUT8 by CHX treatment (A). Relative CD47 expression was measured (B). n = 3; Data are presented as means ± SD. P values were determined by unpaired two-tailed Student's t tests. **C and D,** Immunoblotting of CD47 protein in FUT8-depleted MHCC97-H cells upon MG132, CQ and 3-MA treatment (C). Relative CD47 expression was quantified (D). n = 3; Data are presented as means ± SD. P values were determined by unpaired two-tailed Student's t tests. **E,** Relative CD47 ubiquitination levels of CD47 in CD47 WT or N111Q reconstituted MHCC97-H cells expressing vector, HA-FUT8 WT and HA-FUT8 R365A was quantified. n = 3; Data are presented as means ± SD. P values were determined by unpaired two-tailed Student's t tests.

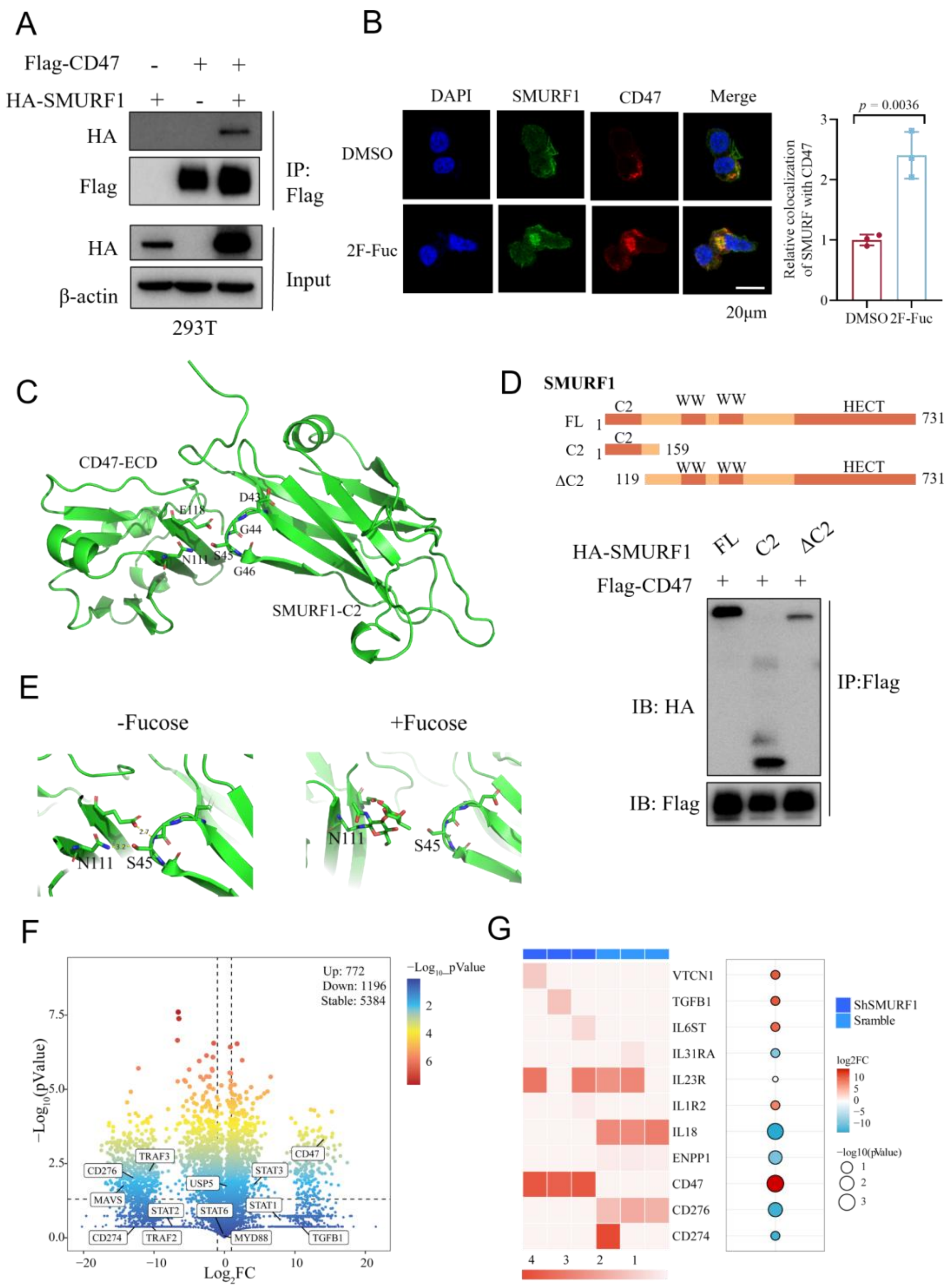

Fig. S6. A, Immunoblotting analysis of CD47 and SMURF1 interaction in 293T cells. **B,** Immunofluorescence of CD47 and SMURF1 interaction in MHCC97-H cells treated with DMSO or 2F-Fuc (100μM). n = 3; Data are presented as means ± SD. P values were determined by unpaired two-tailed Student's t tests. **C,** Molecular simulation analysis of CD47 ECD and SMURF1 C2 domain. **D,** Co-IP analysis of WT CD47 and SMURF1 full length, C2, ΔC2 in 293T cells. **E,** Molecular simulation

analysis of CD47 ECD (with or without core fucose at N111 site) and SMURF1 C2 domain. **F,** Differentially expressed immune-related genes in MHCC97-H cells (ShSMURF1: Scramble, FC = 2, $p < 0.05$). **G,** The expression of immune-related genes in Scramble and ShSMURF1 groups.

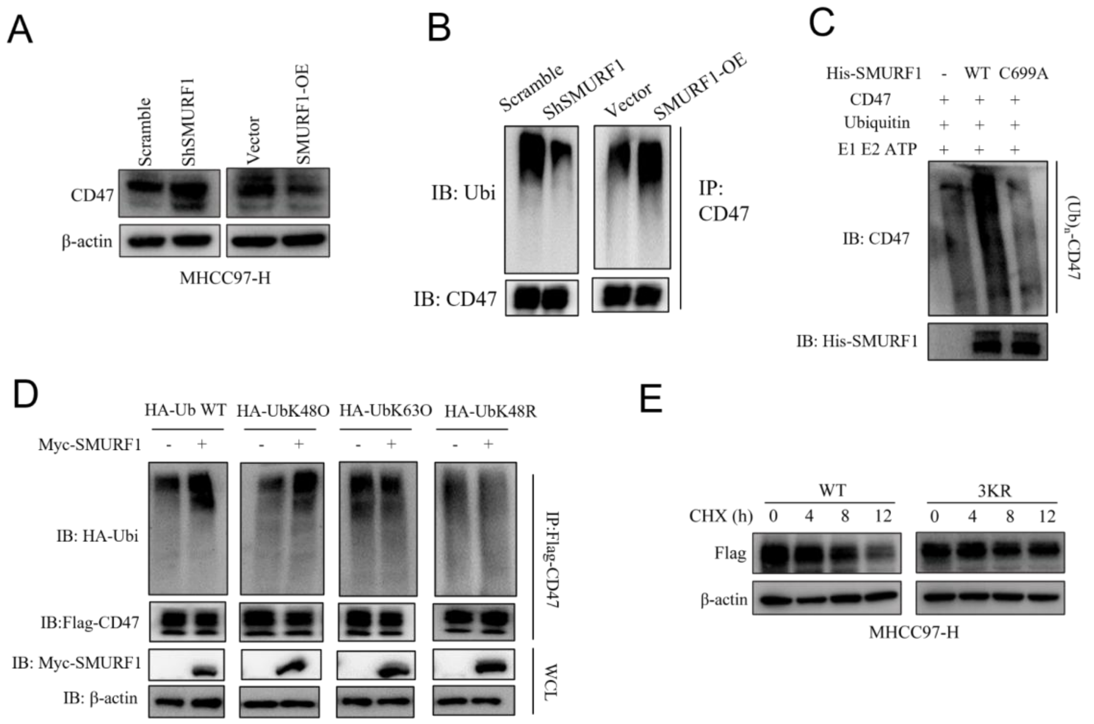

Fig. S7. A, Immunoblotting of CD47 expression in MHCC97-H cells expressing scramble, ShSMURF1, vector and HA-SMURF1. **B,** Immunoblotting analysis of ubiquitination levels of CD47 in MHCC97-H cells expressing scramble, shSMURF1, vector and HA-SMURF1. **C,** In vitro ubiquitination assays of recombinant His-SMURF1 (WT and C699A) and CD47 proteins. **D,** The ubiquitin chain types of CD47 mediated by SMURF1. **E,** Immunoblotting of CD47 WT and 3KR mutant by CHX treatment in MHCC97-H cells.

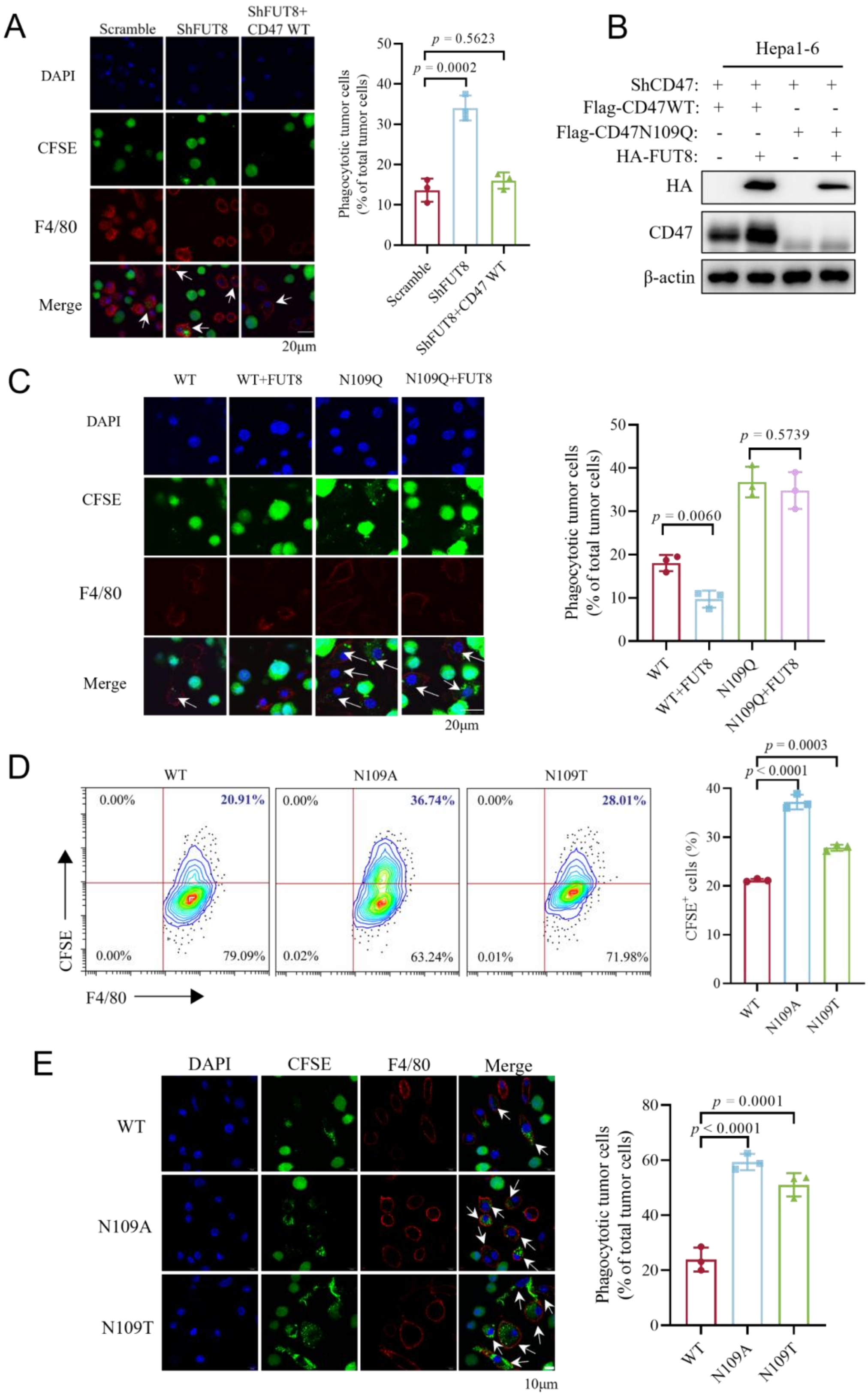

Fig. S8. A, Immunofluorescence analysis of phagocytosis mediated by macrophages co-cultured with Hepa1-6 cells infected with scramble, shFUT8 and shFUT8 reconstituted with CD47 expression. Phagocytosis efficiency was quantified. n = 3;

(Scale bar, 20 μm.) Data are presented as means ± SD. P values were determined by unpaired two-tailed Student's t tests. **B,** Generation of WT or N109Q CD47 reconstituted Hepa1-6 cells expressing vector and HA-FUT8. **C,** Immunofluorescence analysis of phagocytosis analysis of macrophages co-cultured with CD47 WT or N109Q reconstituted Hepa1-6 cells infected with vector and HA-FUT8. Phagocytosis efficiency was quantified. n = 3; (Scale bar, 20 μm.) Data are presented as means ± SD. P values were determined by unpaired two-tailed Student's t tests. **D,** Flow cytometry analysis of BMDM-mediated phagocytosis of N109A, N109T CD47 reconstituted Hepa1-6 cells. n=3; Data are presented as means ± SD. P values were determined by unpaired two-tailed Student's t tests **E,** Immunofluorescence analysis of phagocytosis mediated by macrophages co-cultured with N109A, N109T CD47 reconstituted Hepa1-6 cells. Phagocytosis efficiency was quantified. n = 3; Data are presented as means ± SD. P values were determined by unpaired two-tailed Student's t tests.

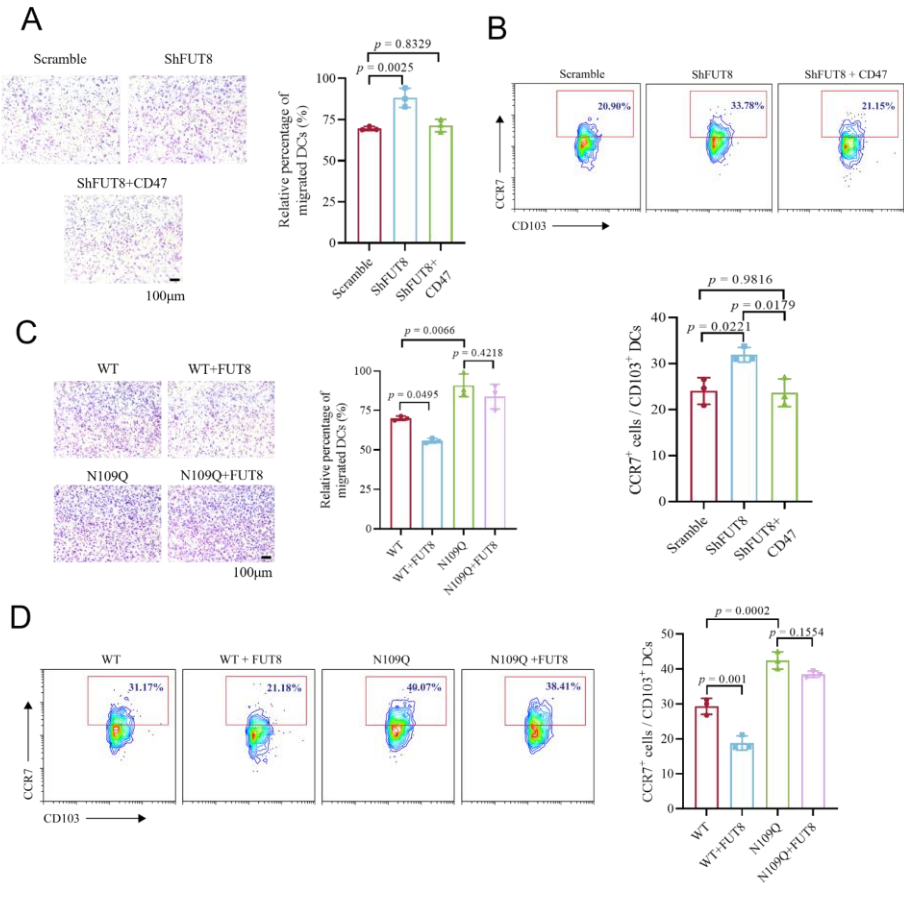

Fig. S9. A, Transwell chemotaxis assays of BMDCs co-cultured with Hepa1-6 cells infected with scramble, shFUT8 and shFUT8 reconstituted with CD47 expression. n = 3; **B,** CCR7 expressoin of CD103^{+} BMDCs co-cultured with Hepa1-6 cells infected with scramble, shFUT8 and shFUT8 reconstituted with CD47 expression. n = 3; Data are presented as means ± SD. P values were determined by unpaired two-tailed Student's t tests. **C,** Transwell chemotaxis assays of BMDCs co-cultured with CD47 WT or N109Q reconstituted Hepa1-6 cells infected with vector and HA-FUT8. n = 3; Data are presented as means ± SD. P values were determined by unpaired two-tailed Student's t tests. **D,** CCR7 expressoin of CD103^{+} BMDCs co-cultured with CD47 WT or N109Q reconstituted Hepa1-6 cells infected with vector and HA-FUT8. n = 3; Data are presented as means ± SD. P values were determined by unpaired two-tailed Student's t tests.

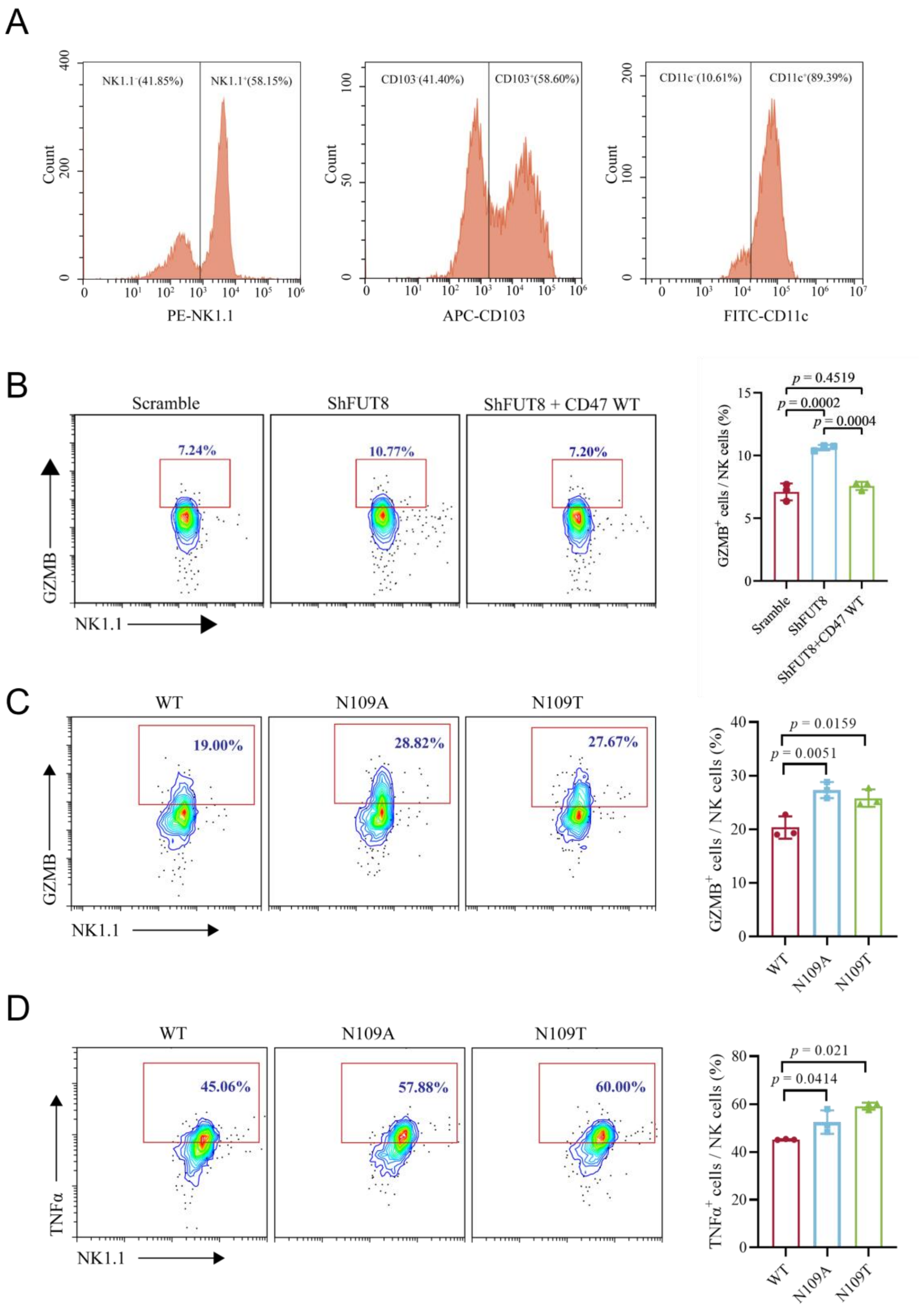

Fig.S10. A, NK cells and CD11c^{+}CD103^{+} DCs respectively isolated from mouse spleen and marrow bone. **B,** Flow cytometry analysis of GZMB production in NK cells cultured with Hepa1-6 cells infected with scramble, shFUT8 and shFUT8 reconstituted with CD47 expression. n = 3; **C,** Flow cytometry analysis of GZMB

production in NK cells co-cultured with WT, N109A, N109T Hepa1-6 cells. n = 3; **D,** Flow cytometry analysis of TNF-α production in NK cells co-cultured with WT, N109A, N109T Hepa1-6 cells. n = 3; These data are depicted as means ± SD. P-values were calculated by unpaired two-tailed Student's t-tests.

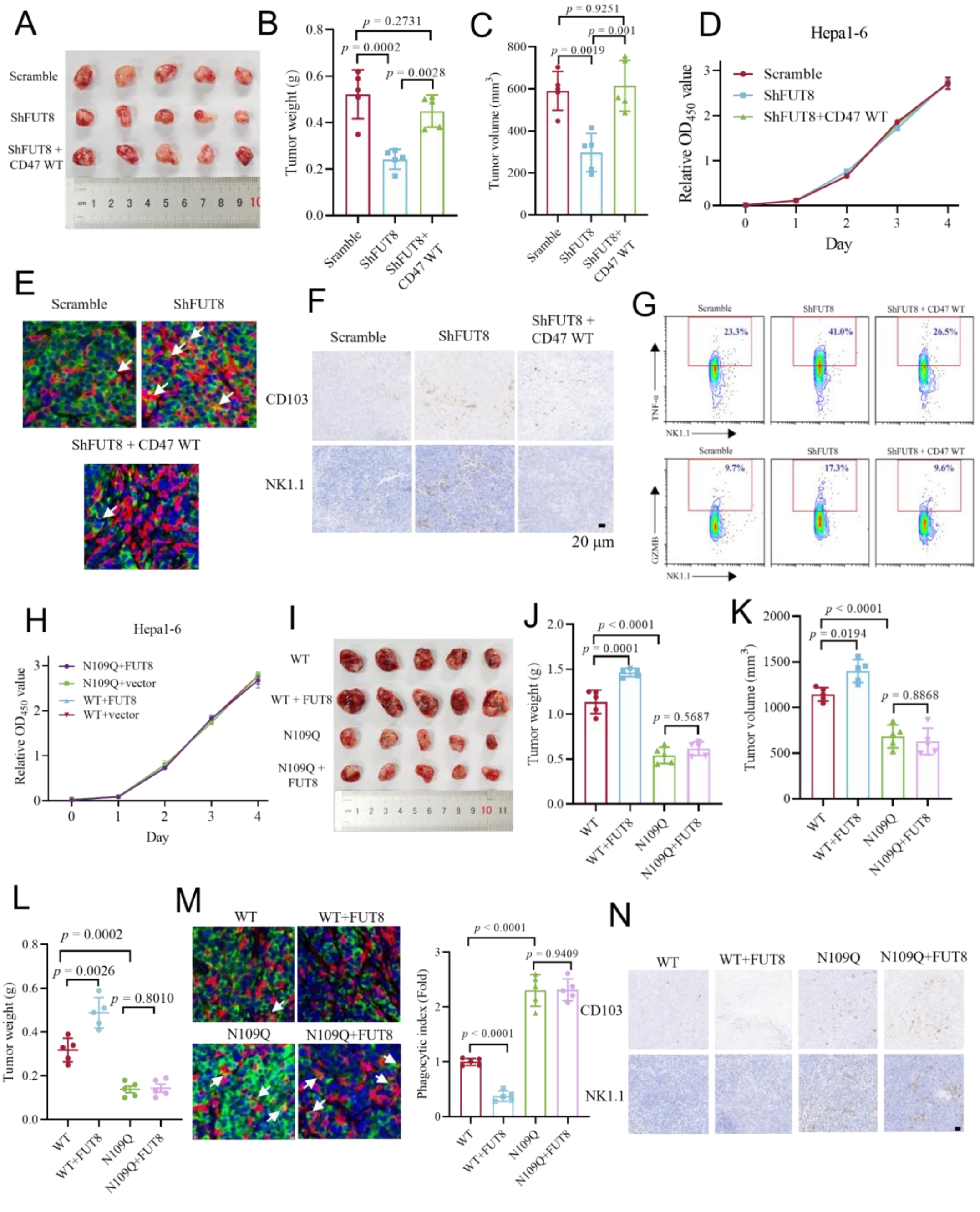

Fig.S11. A-C, Subcutaneous tumor formation in C57BL/6J mice from Hepa1-6 cells infected with scramble, shFUT8 and shFUT8 reconstituted with CD47 expression (A); Tumor weight (B) and volume (C) were calculated. n = 5; Data are presented as

means ± SD. P values were determined by unpaired two-tailed Student's t tests. **D,** Cell proliferation of Hepa1-6 cells infected with scramble, shFUT8 and shFUT8 reconstituted with CD47 expression. **E,** Multiplex immunohistochemical analysis of tumors from Hepa1-6 cells infected with scramble, shFUT8 and shFUT8 reconstituted with CD47 expression. (Scale bar, 20 μm.) **F,** Immunohistochemical analysis of infiltration of $CD103^+$ DCs and $NK1.1^+$ cells in tumors from Hepa1-6 cells infected with scramble, shFUT8 and shFUT8 reconstituted with CD47 expression. (Scale bar, 20 μm.) **G,** Flow cytometry analysis of GZMB and TNFα production of NK cells in tumors from Hepa1-6 cells infected with scramble, shFUT8 and shFUT8 reconstituted with CD47 expression. **H,** Cell proliferation of CD47 WT or N109Q reconstituted Hepa1-6 cells infected with vector and HA-FUT8. **I-K,** Subcutaneous tumor formation in C57BL/6J mice from CD47 WT or N109Q reconstituted Hepa1-6 cells infected with vector and HA-FUT8 (I); Tumor weight (J) and volume (K) were calculated. n = 5; Data are presented as means ± SD. P values were determined by unpaired two-tailed Student's t tests. **L,** Analysis of tumor weight of tumors from CD47 WT or N109Q reconstituted Hepa1-6 cells infected with vector and HA-FUT8. n = 5; Data are presented as means ± SD. P values were determined by unpaired two-tailed Student's t tests. **M,** Multiplex immunohistochemical analysis of tumors from CD47 WT or N109Q reconstituted Hepa1-6 cells infected with vector and HA-FUT8. Phagocytosis efficiency was quantified. n = 5; (Scale bar, 20 μm.) Data are presented as means ± SD. P values were determined by unpaired two-tailed Student's t tests. **N,** Immunohistochemical analysis of infiltration of $CD103^+$ DCs and $NK1.1^+$ cells in tumors from CD47 WT or N109Q reconstituted Hepa1-6 cells infected with vector and HA-FUT8. (Scale bar, 20 μm.) These data are depicted as means ± SD. P-values were calculated by unpaired two-tailed Student's t-tests.

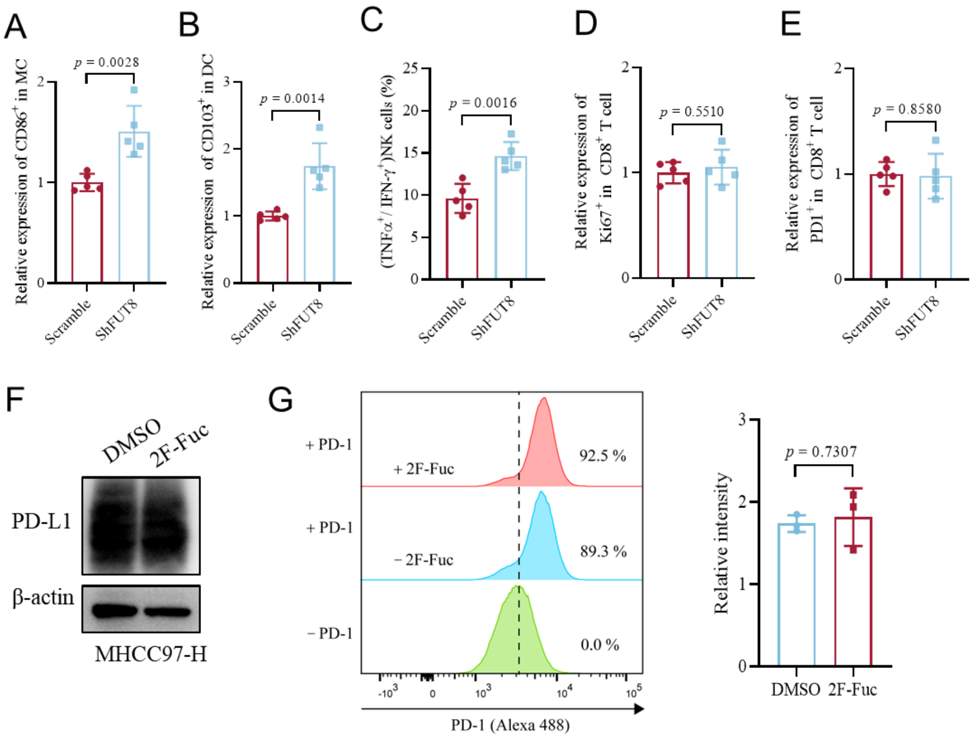

Fig. S12. A-E, Proportion of CD86$^+$ macrophages (A), CD103$^+$ DCs (B), TNFα$^+$/IFNγ$^+$ NK cells (C), Ki67$^+$ (D), and PD1$^+$ CD8$^+$ T cells (E) in sramble and shFUT8 groups by CyTOF. n = 5. Data are presented as means ± SD. P values were determined by unpaired two-tailed Student's t tests. **F,** Immunoblotting of PD-L1 expression in MHCC97-H cells treated with DMSO or 2F-Fuc (100μM). **G,** Flow cytometry of PD-1 binding with PD-L1 on MHCC97-H cells treated with DMSO or 2F-Fuc (100μM). n = 3; Data are presented as means ± SD. P values were determined by unpaired two-tailed Student's t tests.

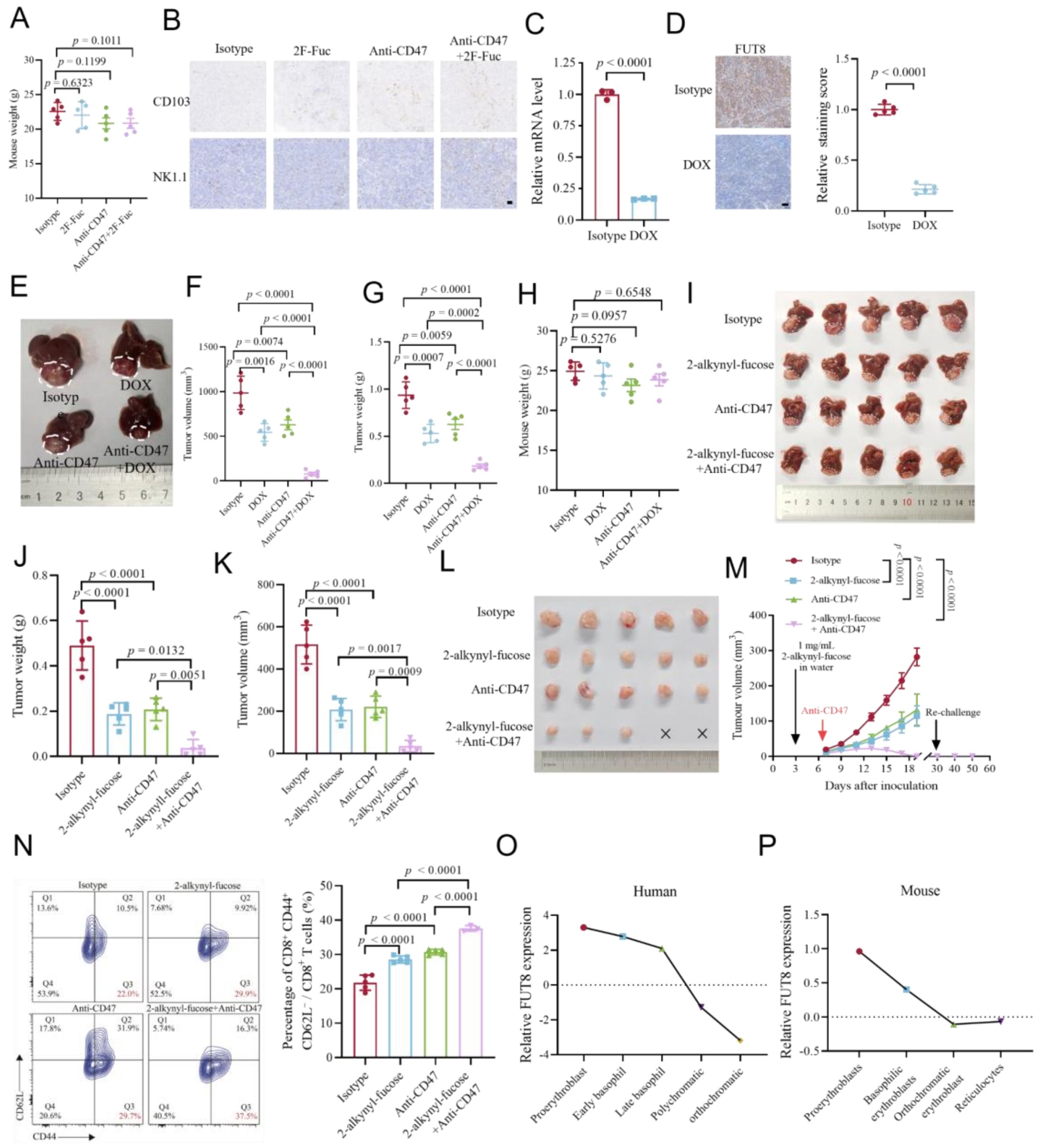

Fig. S13. A, Mouse weight of synergetic therapy with 2F-Fuc treatment and anti-CD47 antibody in HCC orthotopic tumor model. n = 5; Data are presented as means ± SD. P values were determined by unpaired two-tailed Student's t tests. **B,** Infiltration of $CD103^+$ DCs and $NK1.1^+$ cells in tumors from combined therapy with 2F-Fuc treatment and anti-CD47 antibody by immunohistochemical analysis. (Scale bar, 20 μm.) **C,** Quantitative PCR analysis of FUT8 mRNA levels in Hepa 1-6 cells infected with DOX-induced FUT8 knockdown plasmid upon DOX treatment. n = 5; Data are presented as means ± SD. P values were determined by unpaired two-tailed Student's t tests. **D,** IHC staining of tumors from Hepa 1-6 cells infected with DOX-induced FUT8 knockdown plasmid with or without DOX treatment.

Relative staining score was measured. n = 5; (Scale bar, 20 μm.) Data are presented as means ± SD. P values were determined by unpaired two-tailed Student's t tests. **E,** Orthotopic tumor formation in C57BL/6J mice from Hepa1-6 cells infected with doxycycline (DOX)-induced FUT8 knockdown plasmid upon DOX or/and anti-CD47 antibody treatment. **F-H,** Tumor volume (F), tumor weight (G) and mouse weight (H). n = 5; Data are presented as means ± SD. P values were determined by unpaired two-tailed Student's t tests. **I-K,** Orthotopic tumor formation from Hepa1-6 cells in C57BL/6J mice treated with the oral FUT8 inhibitor (2-alkynyl- fucose) or/and anti-CD47 antibody (I); Tumor volume (J) and tumor weight (K). n = 5; Data are presented as means ± SD. P values were determined by unpaired two-tailed Student's t tests. **L and M,** Subcutaneous tumor formation in C57BL/6J mice from Hepa1-6 cells treated with the oral FUT8 inhibitor (2-alkynyl- fucose) or/and anti-CD47 antibody (L); Tumor growth before and after re-challenge was calculated (M)**.** n = 5; Data are presented as means ± SD. P values were determined by unpaired two-tailed Student's t tests. **N,** Flow cytometry of effector memory T cells ($CD8^+$ $CD44^+$ $CD62L^-$) population in the tumor-draining lymph nodes of tumor-bearing mice upon 2-alkynyl- fucose or/and anti-CD47 antibody treatment. n = 5; Data are presented as means ± SD. P values were determined by unpaired two-tailed Student's t tests. **O and P,** Relative FUT8 expression during terminal erythroid differentiation in human (I) and mice (J) based on RNA-seq data.

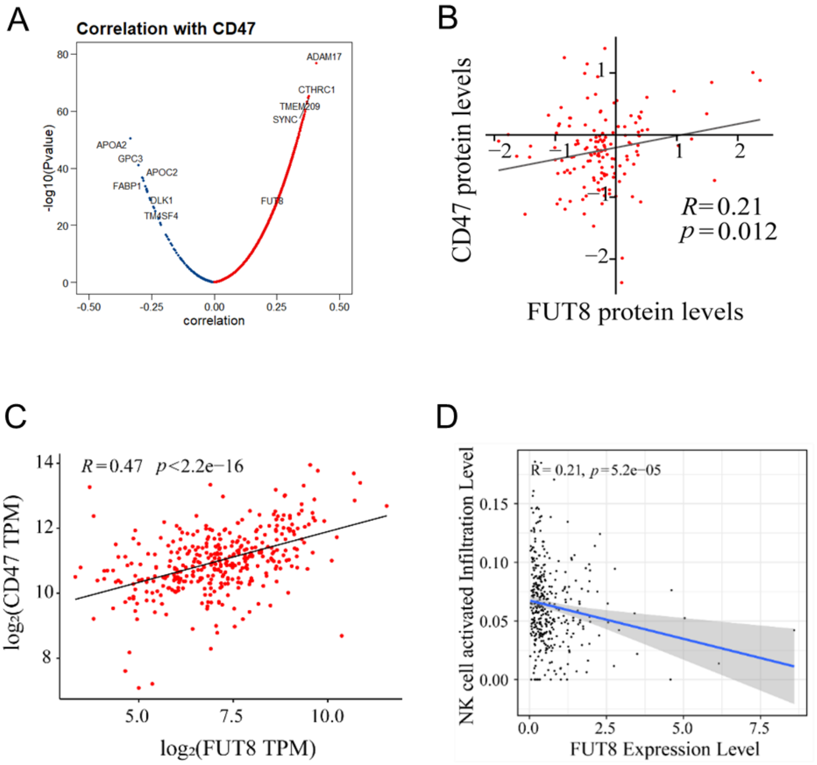

E

Protein	CD276		CD63		IL7R		IL1R1	
	Vector	FUT8-OE	Vector	FUT8-OE	Vector	FUT8-OE	Vector	FUT8-OE
Glycans	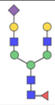		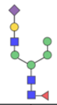		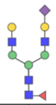		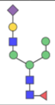	
Intensity	4.77×10^{7}	6.22×10^{7}	1.1×10^{7}	2.62×10^{7}	6.17×10^{7}	8.23×10^{7}	1.10×10^{7}	2.62×10^{7}
Protein	IL6ST		KIAA1549		IL18RAP		SLC39A10	
	Vector	FUT8-OE	Vector	FUT8-OE	Vector	FUT8-OE	Vector	FUT8-OE
Glycans	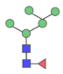		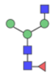		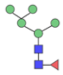		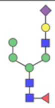	
Intensity	0	0.95×10^{7}	1.27×10^{7}	2.55×10^{7}	0	1.26×10^{7}	0.81×10^{7}	2.15×10^{7}

FigS11. A, Correlation between FUT8 and CD47 mRNA levels from single-cell sequencing datasets (CNP0000650). **B,** Correlation between FUT8 and CD47 protein levels from CPTAC. **C,** Correlation of CD47 and FUT8 mRNA levels from TCGA-LIHC RNA-seq data. **D,** Correlation of FUT8 gene expression and activated NK cell infiltration. These data are depicted as means ± SD. P-values were calculated by unpaired two-tailed Student's t-tests. **E,** Representative core fucosylated N-glycans of 8 immune-related proteins in MHCC97-H cells infected with vector and HA-FUT8.

Supplementary Table 1: Information about antibodies used in this study.

Antibody	Brand	Cat#	Dilution
anti-hCD47	R&D Systems	AF4670-SP	1:1000
anti-mCD47	R&D Systems	AF1866-SP	1:1000
Anti-CD47	Cell Signaling Technology	63000T	1:1000
anti-GFP	Abcam	ab290	1:1000
anti-FUT8	Proteintech	29430-1-AP	1:1000
anti-SMURF1	Abcam	ab300408	1:1000
anti-F4/80	Abcam	ab300421	1:1000
anti-NK1.1	Proteintech	65138	1:1000
anti-Ubi	Abcam	ab137031	1:1000
anti-CD103	Cell Signaling Technology	95835S	1:1000
anti-HA tag	Cell Signaling Technology	3724T	1:1000
anti-β-actin	Cell Signaling Technology	4967S	1:1000
anti-Flag	Abcam	ab125243	1:1000
anti-c-Myc tag	Abcam	ab9132	1:1000

Supplementary Table 2: Sequences of primes used in this study.

Prime	Sequence
β-actin/qPCR/F	CATGTACGTTGCTATCCAGGC
β-actin/qPCR/R	CTCCTTAATGTCACGCACGAT
hCD47/qPCR/F	AGAAGGTGAAACGATCATCGAGC
hCD47/qPCR/R	CTCATCCATACCACCGGATCT
mCD47/qPCR/F	TGCGGTTCAGCTCAACTACTG
mCD47/qPCR/R	ACGATGCAAGGGATGACCAC
hFUT8/qPCR/F	AACTGGTTCAGCGGAGAATAAC
hFUT8/qPCR/R	TGAGATTCCAAGATGAGTGTTCG
mFUT8/qPCR/F	TGATTGCTTATGGCACCCAG
mFUT8/qPCR/R	AGCTCGACCACTTGAATGTTTT
SMURF1/qPCR/F	TGTGAAAAACACATTGGACCCA
SMURF1/qPCR/R	ACGCTAATGGTTATCGAATCCG
CD47 K74R/F	CTCTAAACAGGTCCACTGTCCCCACTGACTTT
CD47 K74R/R	CAGTGGACCTGTTTAGAGCTCCATCAAAGGTG
CD47 K85R/F	GTAGTGCAAGAATTGAAGTCTCACAATTACTAAAAGG AGATGCC
CD47 K85R/R	ACTTCAATTCTTGCACTACTAAAGTCAGTGGGGAC
CD47 K99R/F	CCTCTTTGAGGATGGATAAGAGTGATGCTGTCTC
CD47 K99R/R	CTTATCCATCCTCAAAGAGGCATCTCCTTTTAGTAATTG TGA
CD47 K102R/F	AGATGGATAGGAGTGATGCTGTCTCACACACA
CD47 K102R/R	CATCACTCCTATCCATCTTCAAAGAGGCATCTCC
CD47 K290R/F	TTATATGAGGTTTGTGGCTTCCAATCAGAAGACTATAC AACC
CD47 K290R/R	GCCACAAACCTCATATAAACTAGTCCAAGTAATTGTGC
CD47 N23Q/F	CTACTATTTCAAAAAACAAAATCTGTAGAATTCACG
CD47 N23Q/R	ATTTTGTTTTTTGAAATAGTAGCTGAGCTGAT
CD47 N34Q/F	ACGTTTTGTCAAGACACTGTCGTCATTCCATG
CD47 N34Q/R	ACAGTGTCTTGACAAAACGTGAATTCTACGAG
CD47 N50Q/F	AGGCACAACAAACTACTGAAGTATACGTAAAG
CD47 N50Q/R	TCAGTAGTTTGTTGTGCCTCCATATTAGTAAC
CD47 N73Q/F	GAGCTCTACAAAAGTCCACTGTCCCCACTGA
CD47 N73Q/R	GTGGACTTTTGTAGAGCTCCATCAAAGGTGTA
CD47 N111Q/F	ACACAGGACAATACACTTGTGAAGTAACAGAATT
CD47 N111Q/R	ACAAgGTGTATTGTCCTGTGTGTGAGACAGCATCA
CD47 N206Q/F	TCATTAAAGCAAGCTACTGGCCTTGGTTTAA
CD47 N206Q/R	CAGTAGCTTGCTTTAATGAATATTCACCTGGG
FUT8 C365A/F	CATGTCGCACGCACAGACAAAGTGGGAACAGAAG
FUT8 C365A/R	CTGTGCGTGCGACATGGACTCCAATAACTGGATG
SMURF1 C699A/F	CATACCGCCTTTAACCGGATCGACATTCCACCA
SMURF1	TTAAAGGCGGTATGGGCCTTCGGAAGGTTG

C699A/R	
ShhCD47-#1F:	GATCTCCGATTTGGAGAGTAGTAAGCTCGAGCTTACTACTCTCCAAATCGGATTTTTG
ShhCD47-#1R:	TCGACAAAAATCCGATTTGGAGAGTAGTAAGCTCGAGCTTACTACTCTCCAAATCGGA
ShmCD47-#1F:	GATCGCAGAACTACTTGGATTAGTTCTCGAGAACTAATCCAAGTAGTTCTGCTTTTTG
ShmCD47-#1R:	TCGACAAAAAGCAGAACTACTTGGATTAGTTCTCGAGAACTAATCCAAGTAGTTCTGC
ShhFUT8-#1F:	CCGGGTCTATAATGACGGATCTATACTCGAGTATAGATCCGTCATTATAGACTTTTTG
ShhFUT8-#1R:	AATTCAAAAAGTCTATAATGACGGATCTATACTCGAGTATAGATCCGTCATTATAGAC
ShhFUT8-#2F:	CCGGCGTGGAGTGATCCTGGATATACTCGAGTATATCCAGGATCACTCCACGTTTTTG
ShhFUT8-#2R:	AATTCAAAAACGTGGAGTGATCCTGGATATACTCGAGTATATCCAGGATCACTCCACG
ShmFUT8-#1F:	CCGGCGCAGAATGCAAGTGGATAAACTCGAGTTTATCCACTTGCATTCTGCGTTTTT
ShmFUT8-#1R:	AATTAAAAACGCAGAATGCAAGTGGATAAACTCGAGTTTATCCACTTGCATTCTGCG
ShmFUT8-#2F:	CCGGGCCCATACACAGTACAATAATCTCGAGATTATTGTACTGTGTATGGGCTTTTT
ShmFUT8-#2R:	AATTAAAAAGCCCATACACAGTACAATAATCTCGAGATTATTGTACTGTGTATGGGC
ShSMURF1-#1F:	CCGGCTGGAGGTTTATGAGAGGAATCTCGAGATTCCTCTCATAAACCTCCAGTTTTTG
ShSMURF1-#1R:	AATTCAAAAACTGGAGGTTTATGAGAGGAATCTCGAGATTCCTCTCATAAACCTCCAG
